# Time-Averaged Wavefront Analysis Demonstrates Preferential Pathways of Atrial Fibrillation, Predicting Pulmonary Vein Isolation Acute Response

**DOI:** 10.3389/fphys.2021.707189

**Published:** 2021-09-27

**Authors:** Caroline H. Roney, Nicholas Child, Bradley Porter, Iain Sim, John Whitaker, Richard H. Clayton, Jacob I. Laughner, Allan Shuros, Petr Neuzil, Steven E. Williams, Reza S. Razavi, Mark O'Neill, Christopher A. Rinaldi, Peter Taggart, Matt Wright, Jaswinder S. Gill, Steven A. Niederer

**Affiliations:** ^1^School of Biomedical Engineering and Imaging Sciences, King's College London, London, United Kingdom; ^2^INSIGNEO Institute for In Silico Medicine and Department of Computer Science, University of Sheffield, Sheffield, United Kingdom; ^3^Boston Scientific Corp, St. Paul, MN, United States; ^4^Department of Cardiology, Na Holmolce Hospital, Prague, Czechia; ^5^Department of Cardiology, Guy's and St Thomas' Hospital, London, United Kingdom; ^6^Institute of Cardiovascular Science, University College London, London, United Kingdom

**Keywords:** atrial fibrillation mechanisms, catheter ablation, pulmonary vein isolation, computational modelling, signal processing

## Abstract

Electrical activation during atrial fibrillation (AF) appears chaotic and disorganised, which impedes characterisation of the underlying substrate and treatment planning. While globally chaotic, there may be local preferential activation pathways that represent potential ablation targets. This study aimed to identify preferential activation pathways during AF and predict the acute ablation response when these are targeted by pulmonary vein isolation (PVI). In patients with persistent AF (*n* = 14), simultaneous biatrial contact mapping with basket catheters was performed pre-ablation and following each ablation strategy (PVI, roof, and mitral lines). Unipolar wavefront activation directions were averaged over 10 s to identify preferential activation pathways. Clinical cases were classified as responders or non-responders to PVI during the procedure. Clinical data were augmented with a virtual cohort of 100 models. In AF pre-ablation, pathways originated from the pulmonary vein (PV) antra in PVI responders (7/7) but not in PVI non-responders (6/6). We proposed a novel index that measured activation waves from the PV antra into the atrial body. This index was significantly higher in PVI responders than non-responders (clinical: 16.3 vs. 3.7%, *p* = 0.04; simulated: 21.1 vs. 14.1%, *p* = 0.02). Overall, this novel technique and proof of concept study demonstrated that preferential activation pathways exist during AF. Targeting patient-specific activation pathways that flowed from the PV antra to the left atrial body using PVI resulted in AF termination during the procedure. These PV activation flow pathways may correspond to the presence of drivers in the PV regions.

## Introduction

Patients with persistent atrial fibrillation (AF) are a diverse population. Some patients with persistent AF require multiple catheter ablation procedures with more extensive ablation strategies, which may still be ultimately unsuccessful, while for other patients, isolation of the pulmonary veins (PVs) is a sufficient treatment approach (Verma et al., 2015). Identifying appropriate ablation strategies for specific patients with persistent AF, including stratifying patients for whom pulmonary vein isolation (PVI) will be sufficient treatment, remains a clinical challenge (Johner et al., 2019). If solved, this could lead to improved safety and better patient selection, as well as decreased time and cost for procedures. Determining the optimal catheter ablation therapy for patients with persistent AF requires an understanding of the patient-specific mechanisms that sustain AF (Calkins et al., 2017).

It can be challenging to characterise mechanisms sustaining AF because AF appears chaotic and disorganised (Lee et al., 2014). In particular, the Signal Transfer of Atrial Fibrillation to Guide Human Treatment (STARLIGHT) clinical study, which analysed AF complexity from basket mapping catheters, found no evidence for electrical drivers of persistent AF within the mapping field and instead demonstrated multiple wavelets of activation (Child et al., 2018). In the current study, we hypothesised that while globally chaotic, there may be local preferential activation pathways that can be identified by analysing AF activation sequences probabilistically over time. We further hypothesised that the features of these activation pathways can be used to predict PVI ablation response.

In this study, we aimed to develop a technique for identifying preferential pathways of activation by analysing AF wavefront patterns over time. Then, we aimed to use this information to predict PVI acute ablation response, with the hypothesis that in cases where PVI terminated AF during the procedure, the source of preferential pathways, whether re-entrant or focal, should originate from the PVs. We performed this analysis on recordings from patients with persistent AF collected using simultaneous biatrial contact mapping with 64 electrode constellation catheters. To test the sensitivity of the algorithm to driver type, catheter size, and catheter contact, we used synthetic signals obtained from AF simulations for a virtual patient cohort in which the underlying AF mechanisms are known.

## Methods

### Clinical Basket Recordings

This study assessed 14 patients with persistent AF from the STARLIGHT clinical trial (NCT01765075) (Child et al., 2018). These patients had a mean age of 61 ± 8 years, mean duration of persistent AF of 20.2 ± 6.7 months, mean left ventricular ejection fraction of 59 ± 10%, mean left atrium (LA) size of 46 × 55 mm, and mean right atrium (RA) size of 42 × 55 mm. Other properties are as follows: five patients had hypertension; three had obstructive sleep apnoea; seven had a body mass index > 30.

All patients gave informed consent and the study was approved by the local ethics committee. Simultaneous biatrial contact mapping was performed with two 64 electrode Constellation catheters (size, 48, 60, and 75 mm; Boston Scientific, Saint Paul, Minnesota), using the Ensite Velocity cardiac mapping system (St. Jude Medical, St. Paul, MN, USA). The recordings were performed pre-ablation, post-PVI, and post each subsequent lesion set. The recordings were sampled at a rate of 2.0345 kHz and the recording duration was in the range from 49.8 to 245 s (mean: 147.4 ± 74.6 s). Acute PVI responders were patients who went into sinus rhythm or an atrial tachycardia or flutter following PVI, while acute PVI non-responders remained in AF. Seven of the patients were PVI responders, six were non-responders to PVI, and one patient presented with atrial tachycardia, which provided data for validating the algorithms on a simpler rhythm. The ablation protocol used isolated the PV first and secondary lines were only applied if the patient remained in AF after PVI.

### Simulation Data: Constructing a Virtual Patient Cohort

Imaging data for 25 patients with persistent AF were used to construct a virtual patient cohort; ethical approval was granted by the regional ethics committee (17/LO/0150 and 15/LO/1803). This dataset was a separate clinical imaging cohort from the STARLIGHT cohort. These cases have a range of atrial sizes from 90.8 to 244.9 cm^2^ (mean 143.4 ± 30.5 cm^2^) and LA fibrosis surface areas ranging from 5.16 to 46.3 cm^2^ (mean 20.7 ± 13.5 cm^2^). We combined different anatomies with different fibrosis maps from the dataset to create a virtual cohort of patients, covering the range of atrial sizes, morphologies, anisotropies, and fibrosis distributions that were seen in patients with AF (Sim et al., 2019). AF simulations for these models have different underlying AF mechanisms, cycle lengths, and arrhythmia complexities. As such, we tested PVI response across a range of different structural and electrical AF properties.

For each case, the left atrium was segmented from contrast-enhanced magnetic resonance angiogram (CE-MRA) scans and registered with late-gadolinium enhancement magnetic resonance imaging (LGE-MRI) scans using CEMRGApp software (https://cemrg.com/software/cemrgapp.html) King's College London, London, UK (Razeghi et al., 2020). Segmented meshes were re-meshed to produce a regular edge length of 0.34 mm, using mmgtools software (www.mmgtools.org) (Dapogny et al., 2014). Simulations were run for monolayer left atrial models using the Cardiac Arrhythmia Research Package (CARPentry) simulator, with the monodomain model for excitation propagation and the Courtemanche-Ramirez-Nattel human atrial ionic model (Courtemanche et al., 1998), and with modifications to represent the effects of AF electrical remodelling (Courtemanche et al., 1999; Vigmond et al., 2003). Longitudinal conductivity was assigned as 0.4 S/m and transverse as 0.1 S/m (Bayer et al., 2016). Models were constructed with repolarization heterogeneity by labelling each PV and the LA appendage using Paraview software, Kitware (https://www.paraview.org/) and assigning different ionic conductances, following our previous studies (Roney et al., 2018, 2019). Fibrotic remodelling was incorporated in each mesh according to the LGE-MRI intensity values, which were assigned as the maximum value through the wall (Sim et al., 2019).

Fibrotic effects were incorporated as regions of conduction slowing [100% conduction velocity (CV) in regions of 0–56% normalised LGE intensity, 80% CV for 56–60% LGE; 60% CV for 60–64% LGE, and 40% CV for > 64% normalised LGE intensity]. The ionic properties were also modified in fibrotic regions to represent the effects of elevated TGF-β1 (maximal ionic conductances were rescaled in regions with LGE intensity > 3 standard deviations above the mean of the blood pool as follows: 50% gK1, 60% gNa, and 50% gCaL) (Roney et al., 2016; Zahid et al., 2016; Krueger et al., 2014).

Simulations were run for each anatomy, with or without patient-specific fibrosis and with two further different fibrosis maps randomly selected from the remaining 24 fibrosis maps to create a virtual patient cohort of 100 models. These 100 models were used for testing the preferential pathways analysis algorithms. Fibrosis distributions were mapped between atrial anatomies by expressing all anatomies in universal atrial coordinates, following our previous methodology (Roney et al., 2019).

To investigate the effects of fibre field on simulated preferential pathways and PVI acute response, fibrosis model simulations with three different fibre fields were compared. The baseline fibre field incorporated in all model set-ups (the 100 models described above) was the rule-based LA endocardial fibre field in the study of Labarthe et al. (2014). Additional simulations were performed for the 25 different anatomies incorporating patient-specific fibrosis with a diffusion tensor MRI (DTMRI) human atrial fibre field (dataset number 1 from Roney et al., 2020b) or an average LA endocardial field constructed from seven DTMRI datasets (Roney et al., 2020b available to download at https://zenodo.org/record/3764917). For each case, atrial fibres were mapped to each atrial mesh using the universal atrial coordinate system (Roney et al., 2019).

### Simulation Data: AF Initiation, Post-processing, and Modelling PVI Ablation

**Figure 1:**
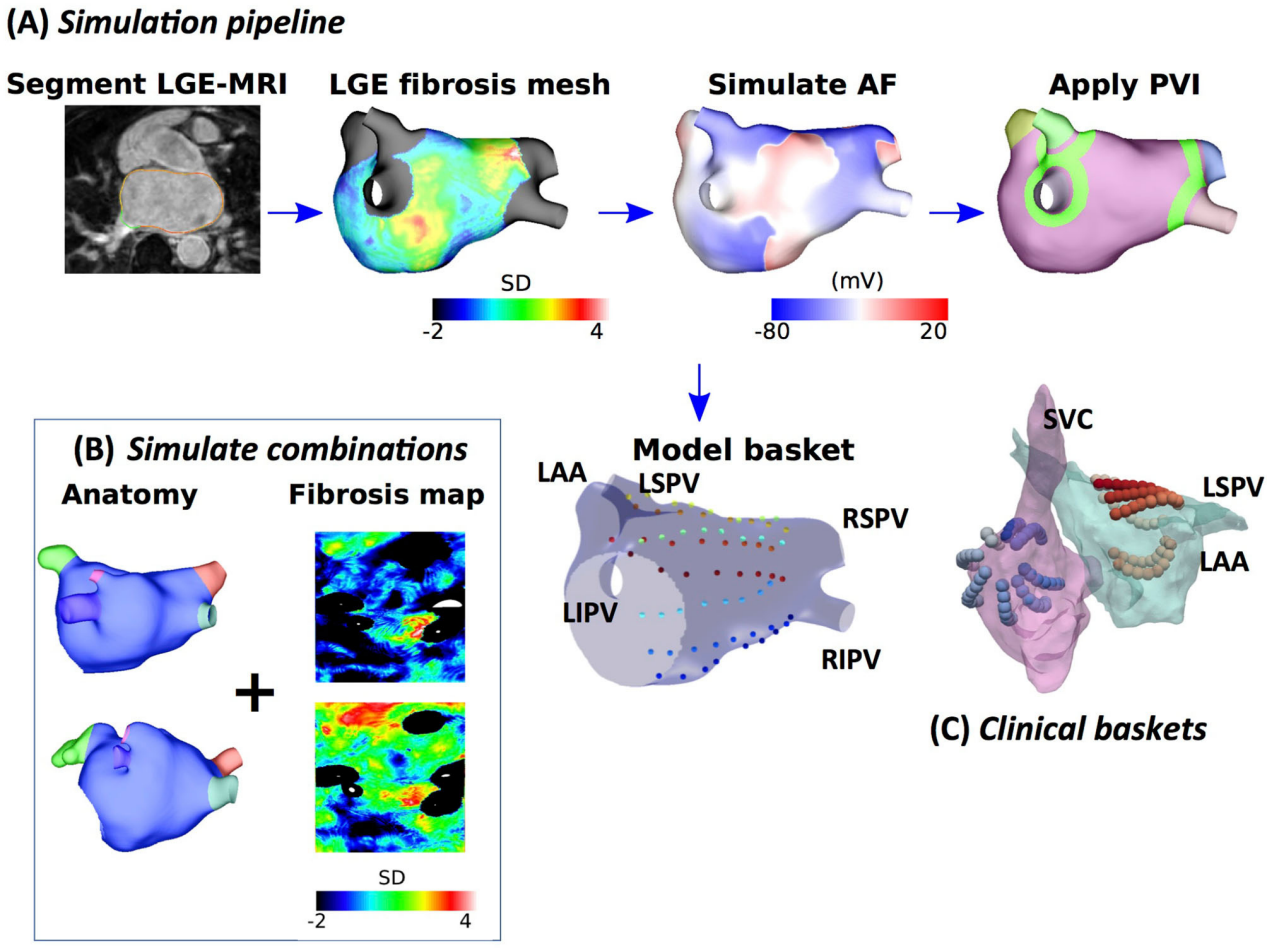
Schematic showing the steps in model construction, simulation, and post-processing. **(A)** Imaging data were segmented, and simulation meshes were created incorporating conductivity and ionic changes in areas of fibrotic tissue identified using LGE-MRI intensity values. Atrial fibrillation (AF) was simulated and analysed at a basket arrangement of points modelled based on clinical electrode locations. Pulmonary vein isolation (PVI) was applied after 5 s. **(B)** Models were generated for different combinations of anatomy and fibrosis distribution to build a virtual patient cohort of 100 models. **(C)** Simultaneous biatrial contact mapping was performed with two 64 electrode Constellation catheters.

Atrial fibrillation was induced through burst pacing, and basket catheter electrode signals were simulated across the atria, as shown in Figure 1. For each model, AF was induced through burst pacing the right superior pulmonary vein (RSPV) at a cycle length of 155 ms for five beats following sinus rhythm (Roney et al., 2018). To investigate the effects of AF initiation pacing protocol on preferential pathways and PVI outcome, we considered two additional pacing protocols for the 25 models with patient-specific fibrosis and the rule-based LA endocardial fibre field (Roney et al., 2020a). These methods and results are presented in detail in the Supplementary Material.

Atrial fibrillation transmembrane potential data were analysed at points that correspond to a constellation basket catheter configuration, using the same methodology that was used for the clinical data. Our previous study compared simulated unipolar electrogram phase and bipolar electrogram phase to action potential phase to demonstrate a good agreement (phase singularity trajectory distance < 0.8 mm) (Roney et al., 2017b). To construct a basket arrangement of points in each anatomy, the recording locations from one of the clinical cases were aligned with a simulation mesh such that the largest separation between splines was across the mitral valve annulus (see Figure 1). These locations were then rescaled by two scaling factors to represent a larger and smaller basket and assigned to the closest points on the atrial geometry. Finally, to transfer the basket electrode locations to each atrial geometry, electrode locations were expressed in universal atrial coordinates (Roney et al., 2019) and mapped to the corresponding atrial coordinates on the target anatomy.

Pulmonary vein isolation, which was modelled as two non-conducting rings (tissue conductivity of 0.001 S/m) around the left and right LA-PV junctions, was applied 5 s post-AF initiation for all AF simulations. Pulmonary vein isolation outcome was visually classified at 2 s post-PVI as responder (macro-reentry or termination) or non-responder (AF continues).

### Simulation Data: Biatrial Bilayer Simulations of Different Atrial Rhythms

To test the preferential pathways methodology on different atrial rhythms, we used simulation data from a previously published biatrial bilayer model (Labarthe et al., 2014). Left and right atrial baskets were positioned in each atrium by rescaling, rotation, and translation of clinical basket electrode locations. Atrial flutter was initiated by applying a line stimulus from the tricuspid valve to the inferior vena cava and temporarily adding a line of the block along the crista terminalis. After five re-entry circuits, this line of the block was removed and the re-entry was sustained. The focal activity was simulated by stimulating a region on the posterior wall of the left atrium at a cycle length of 200 ms.

### Electrogram Processing

Unipolar electrogram signals were processed to calculate a normalised derivative signal and a phase signal using a sequence of steps, shown in Supplementary Figure 1. First, QRS subtraction was applied to unipolar electrograms to remove any ventricular artefacts from the signals (Shkurovich et al., 1998). Following QRS subtraction, electrograms were differentiated and the derivative signal was filtered using a sequence of filters typically used prior to dominant frequency analysis to make the signal more sinusoidal (Ng et al., 2007). For each of the 64 electrodes on each basket catheter, filtered derivative signals were normalised and the unipolar phase was also calculated, following our previously validated methodology (Roney et al., 2017b) (for further details see the Supplementary Material). The mean cycle length (CL) was calculated for each electrogram by taking the mean of all the time intervals between peaks of the normalised filtered derivative signal. The mean of all LA or RA electrogram CLs was calculated to give the mean LA or RA CL for each patient.

**Figure 2:**
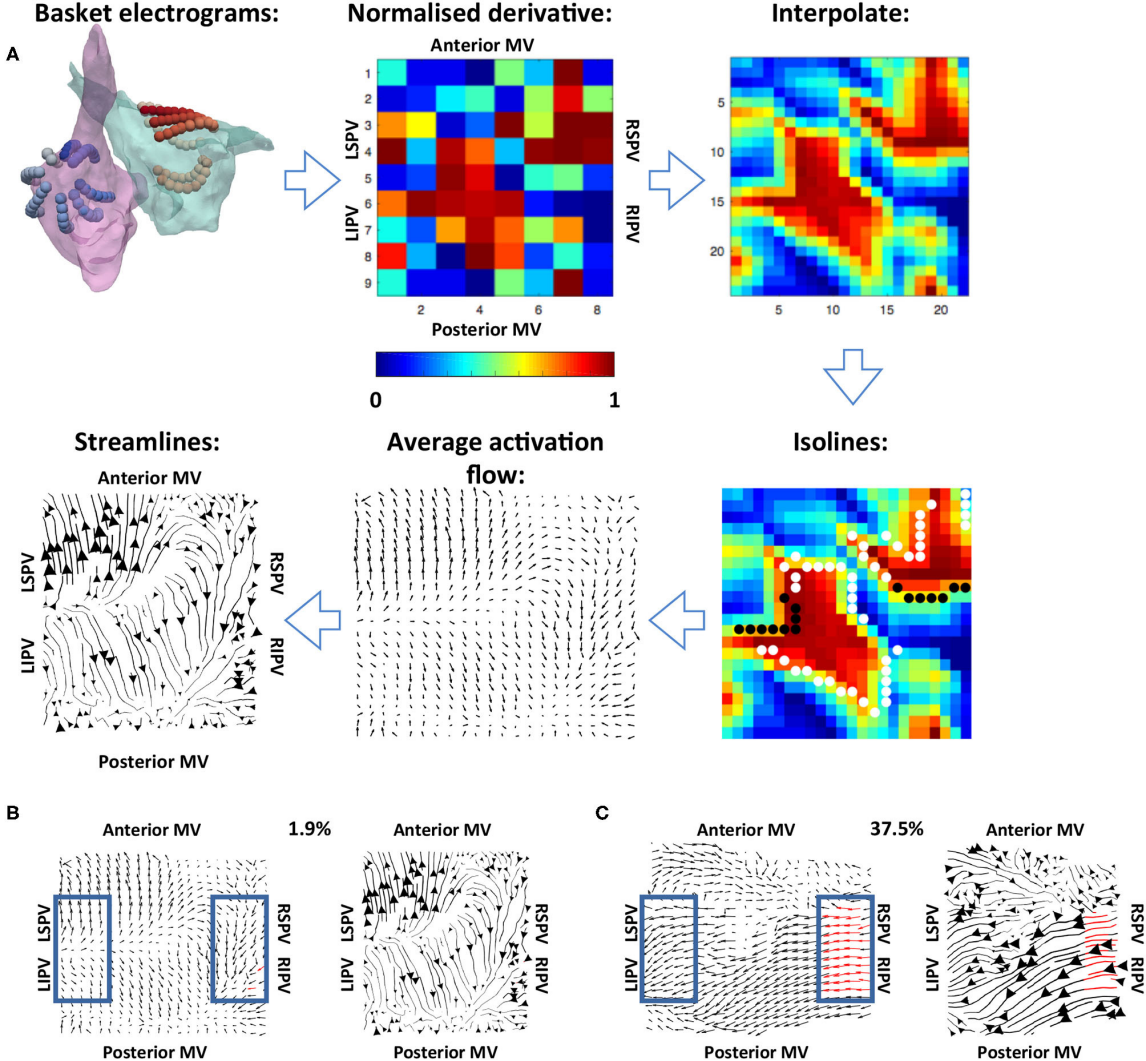
Methodology used for calculating the average activation flow vector field from unipolar electrograms. **(A)** The normalised filtered derivative signal for each electrode on the basket catheter was displayed in a regular 9 × 8 grid. These signals were interpolated to a higher resolution grid, and optical flow vectors were calculated. The vectors were averaged over time for isolines of constant normalised filtered derivative signal (indicated by white and black dots; wavefront and wave back). Finally, streamlines were calculated from average optical flow vectors to visualise activation pathways. The normalised derivative, interpolate and isoline plots are snapshots representing the wavefronts at a given time in the recording, while the average activation flow and streamlines are calculated as an average across the 10 s recording. **(B)** Example PV activation flow calculation for a case with low PV activation flow. Vectors within the blue boxes were assessed, with red vectors tagged as PV antra to LA activation flow. **(C)** Example PV activation flow calculation for a case with high PV activation flow.

Normalised filtered derivative signals were then displayed in a 9 × 8 arrangement corresponding to the eight splines of the basket catheter, which each have eight electrodes, with the anterior mitral valve (MV) spline duplicated at the posterior MV side of the grid, following the study of Child et al. (2018) (see Figure 2). The visualisation used has the posterior MV at the bottom of the grid, the anterior MV at the top, and the left PV (lateral wall) and right PV (septal wall) on the left and right of the grid, respectively. Correspondingly for the right atrium (RA), the inferior vena cava (IVC) is displayed at the bottom of the grid, the superior vena cava (SVC) at the top, the septal tricuspid valve (TV) on the left, and the lateral TV on the right. This 2D arrangement was performed on a case-by-case basis depending on the spline arrangement in relation to the MV and TV location. Finally, these data were linearly interpolated to a higher resolution grid with an additional two points introduced between every two points on the original grid (Roney et al., 2017a).

### Vector Mapping, Streamline Visualisation, and Statistics Over Time

To track the direction of propagation of activation wavefronts, the optical flow of the interpolated grid of normalised filtered derivative signals was calculated. Specifically, a displacement vector was calculated for each normalised filtered derivative pixel at each time frame analysed to show where that pixel is found in the next time frame, i.e., the direction of flow. This was performed at an increment of 40 frames (approximately 20 ms) to allow for sufficient movement of pixels between frames. This implementation followed Horn and Schunck (1981). These optical flow vectors were then averaged for activation wavefronts, which were identified as isolines of 0.9 normalised filtered derivative with greater than three connected pixels (Kay and Gray et al., 2005), as shown in Figure 2. This averaging was performed over 10 s windows using vector addition.

For visualisation of these average optical flow vectors and identification of preferential pathways, activation streamlines were constructed. Activation streamlines were calculated using an adaptation of the technique as proposed in the study of Saliani et al. (2019). Specifically, a Delaunay triangulation of the recording points was calculated to construct a mesh, and streamlines were initially calculated from seeds at each mesh element. This construction progressed both forward and backward along the vector field direction, subject to an angular stopping criterion of 0.7 radians. Finally, a set of these streamlines was built iteratively at the desired spacing by adding streamlines to the set by order of decreasing streamline length subject to a minimum distance criterion (0.5 pixels). Each streamline is plotted with thickness dependent on the magnitude of the underlying average vector field to indicate how often a direction is repeated. The direction of the centre of each streamline is marked with an arrow, again with magnitude proportional to the magnitude of the average vector field at that point. An example is shown in the final subplot of Figure 2.

To quantify the degree of PV antra to LA body activation flow, the percentage of optical flow vectors that were of threshold magnitude and directed into the LA were calculated. This analysis was performed for vectors within a box close to the left PV and a box close to the right PV, indicated in blue in Figures 2B,C. Specifically, vectors within these boxes were tagged as PV to LA activation flow in the case that their magnitude was larger than the mean magnitude across the array to represent a degree of repeatability, and that their direction was within a 90° range into the body. These vectors were identified for both the left PV and the right PV antra, and an activation flow metric was calculated for each box separately as the percentage of all vectors in the boxes tagged as representing PV to LA activation flow. The PV activation flow metric was then calculated as the maximum of the left PV and right PV activation flow metrics. To compare PV activation flow metrics between PVI responders and PVI non-responders, we performed the Wilcoxon signed-rank test and calculated the following metrics: sensitivity; specificity; area under the receiver operating characteristic curve.

Calculation of vector maps and activation flow maps was performed blinded to acute PVI response.

## Results

### Testing on Simulated Atrial Re-entry and Focal Activation

**Figure 3:**
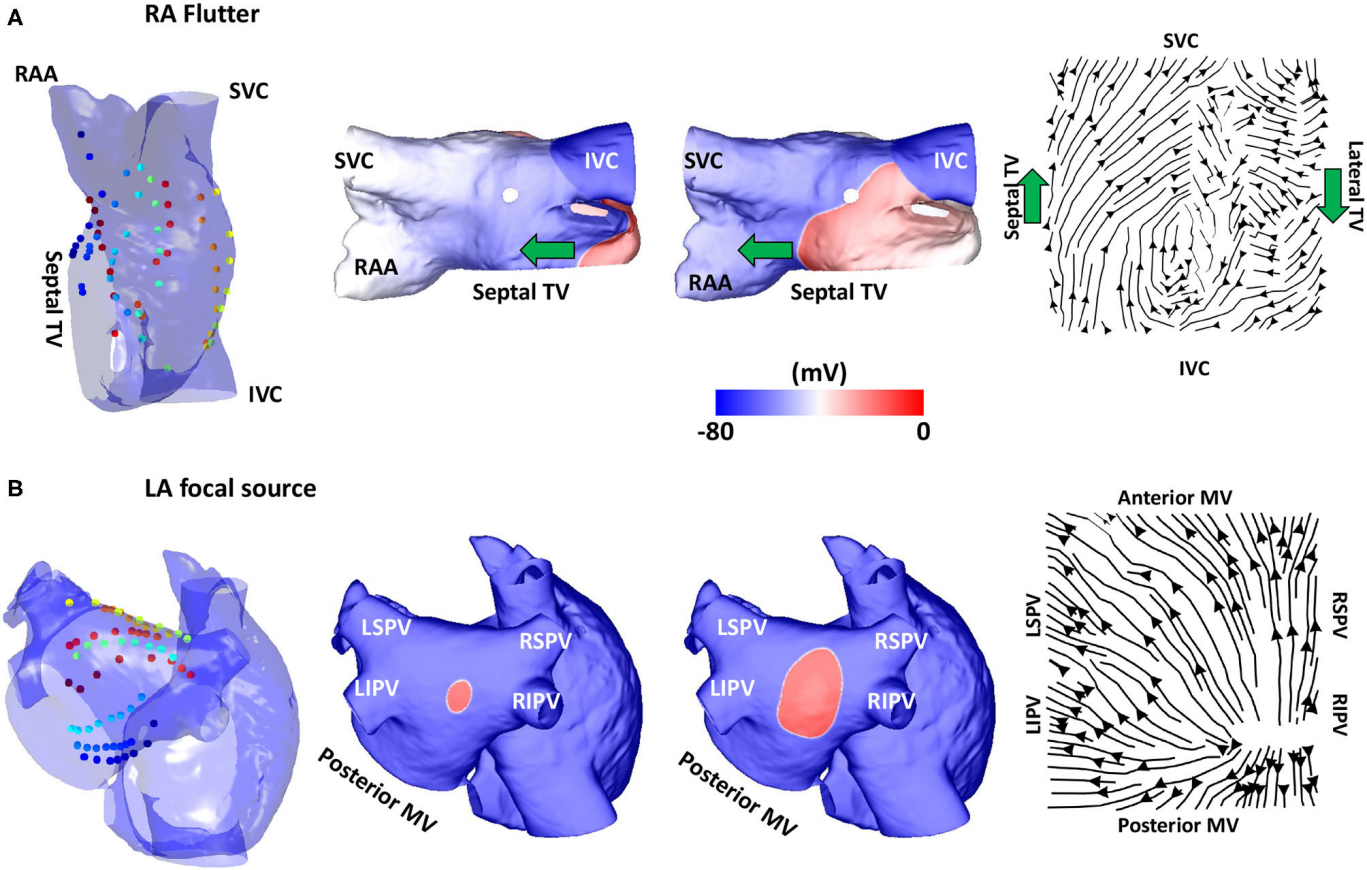
Validation using simulated data: flutter and focal activity. **(A)** Simulated atrial flutter with activation streamlines from the IVC to SVC along the septal wall and from the SVC to IVC along the lateral wall, to complete the re-entry circuit. Basket location, isopotential plots and the activation streamlines are shown. The wavefront propagation path direction is indicated by the green arrows. The orientation of the RA in each sub-figure was chosen to offer easier visualisation of the electrode locations and the wavefront propagation direction. **(B)** A simulated repetitive focal source manifests as a divergent activation streamline field. TV, tricuspid valve; SVC, superior vena cava; IVC, inferior vena cava; RAA, right atrial appendage; MV, mitral valve; LSPV, left superior pulmonary vein; LIPV, left inferior pulmonary vein; RIPV, right inferior pulmonary vein; RSPV, right superior pulmonary vein; LA, left atrium; RA, right atrium.

Simulations of atrial re-entry and focal activation resulted in activation flow patterns that qualitatively reflected the underlying activation, as shown in Figure 3 for the biatrial bilayer model. Simulation data corresponding to an RA atrial flutter is shown in Figure 3A with wavefront propagation from the IVC along the septal wall to the RAA and SVC, which then propagated along the lateral wall from the RAA and the SVC to the IVC. This wavefront propagation, from the IVC to SVC along the septal wall and from the SVC to IVC along the lateral wall, has formed a re-entrant circuit.

The activation streamlines constructed from the average optical flow field vector map show this activation pattern, with the wavefront propagation direction indicated by the green arrows. A re-entrant pattern is visible on the roof of the right atrium because there is a driving pattern around the TV that propagated along the septal wall from the IVC to SVC, and then along the lateral wall from the SVC to the IVC. This resulted in the discontinuity in the streamlines along the roof as one end of the reentrant wavefront travels along the roofline and did not propagate to the other wall as it is still refractory from the previous propagation. A further simulation example representing a fixed focal source is shown in Figure 3B, with an activation streamline map that is divergent from the source location. This demonstrated that the methodology correctly identifies re-entrant and focal mechanisms.

### Testing on Clinical Atrial Tachycardia Data

**Figure 4:**
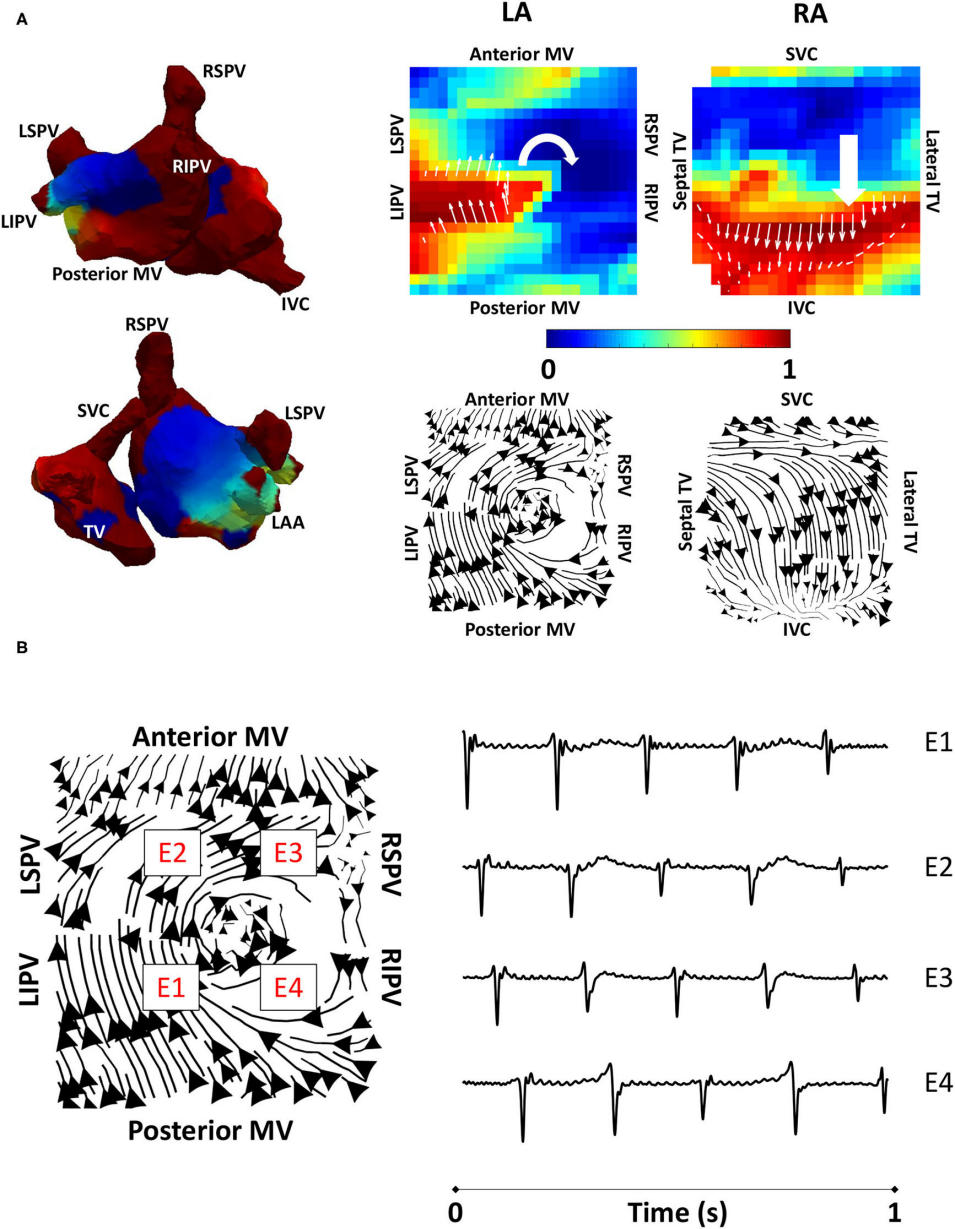
Algorithm validation using clinical atrial tachycardia data. **(A)** Clinical atrial tachycardia phase maps, exhibiting re-entry on the LA posterior wall and passive RA activation are shown on the left of the figure. Normalised filtered derivative signals interpolated to a regular grid are shown on the top right for a single time point to show LA re-entry. Streamlines constructed from the average optical flow activation field for this clinical atrial tachycardia case demonstrate a re-entrant activation pattern on the LA posterior wall, and regular RA activation starting at the RA septal wall as it is activated from the LA. **(B)** Four LA basket electrograms are shown at the grid locations indicated by boxes with E1 to E4 on the streamline map. Locations E1-E4 are sequentially activated, demonstrating the presence of reentry. TV, tricuspid valve; SVC, superior vena cava; IVC, inferior vena cava; RAA, right atrial appendage; MV, mitral valve; LSPV, left superior pulmonary vein; LIPV, left inferior pulmonary vein; RIPV, right inferior pulmonary vein; RSPV, right superior pulmonary vein; LA, left atrium; RA, right atrium.

To validate the developed algorithms on clinical data, we applied the techniques to a typical clinical tachycardia case. Macro-reentrant tachycardia generated a regular activation, which should manifest as clear lines following the activation path, providing suitable data for validating our algorithms. Clinical atrial tachycardia data for one patient with re-entry on the posterior LA wall and passive RA activation, previously analysed in the study of Child et al. (2018), were used to test our preferential pathways methodology. Figure 4A shows phase maps for the unipolar electrogram recordings, together with the normalised filtered derivative signals interpolated to a regular grid on which re-entry on the posterior LA wall is observed and regular passive RA activation. Streamlines that were constructed from the average optical flow activation vector fields for this case had demonstrated a re-entrant activation pattern on the LA posterior wall and regular RA activation starting at the septal RA wall following activation from the LA, offering testing of the technique. Figure 4B shows LA basket electrograms at four locations indicated by the locations E1–E4. These electrograms are sequentially activated, demonstrating the presence of re-entry.

### Testing on Simulated AF Data: Pathway Analysis and PV Activation Flow Metrics for PVI Responders vs. Non-responders

**Figure 5:**
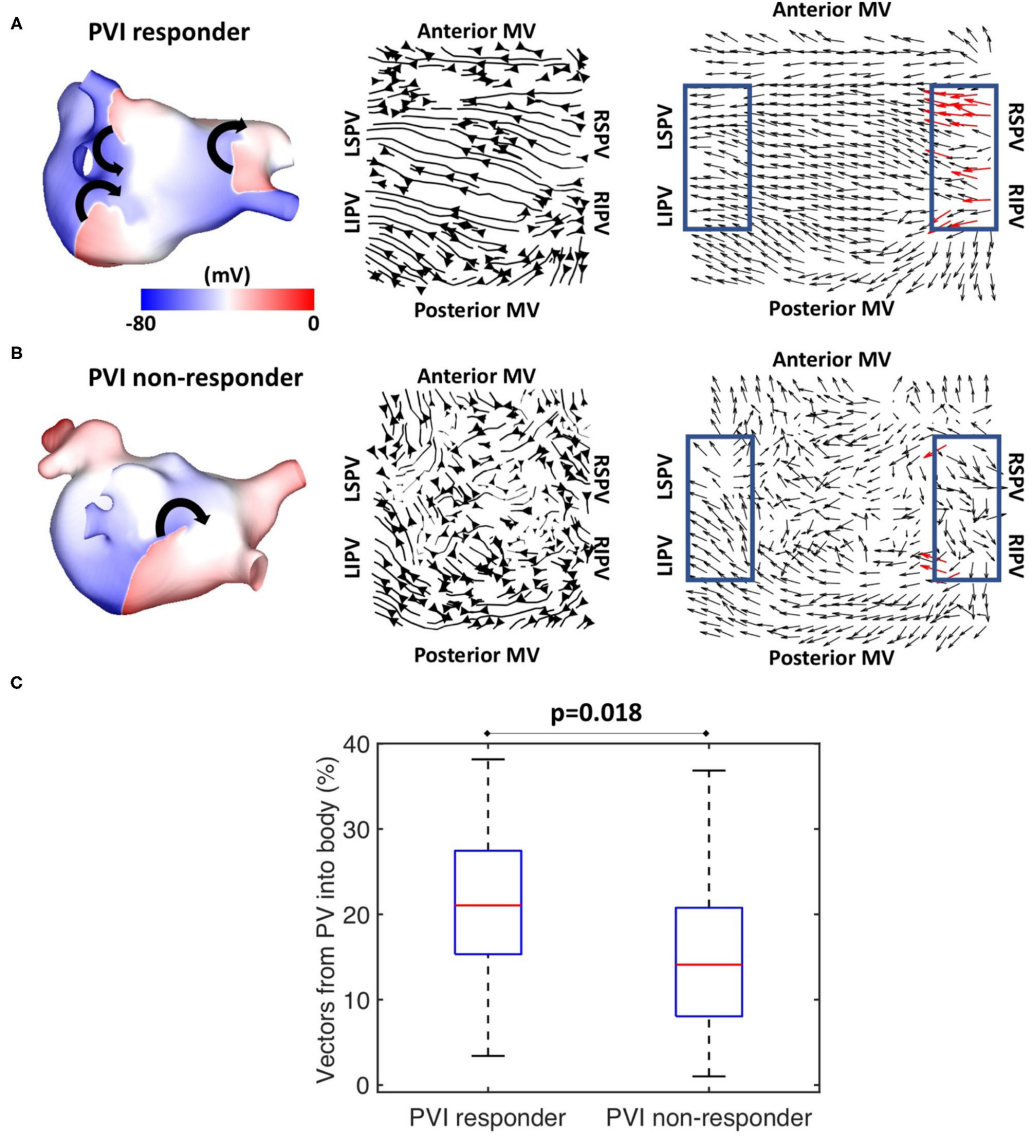
Simulated data PV activation flow metric is significantly different for PVI responder and non-responder cases. Isopotential snapshots (left), streamlines (middle), and optical activation flow (right) plots for examples of **(A)** PVI responder and **(B)** PVI non-responder. PV activation flow was quantified as % of vectors pointing into the LA body in a 90° range within the PV regions (blue boxes). PV activation flow vectors are shown in red. **(A)** For this simulation, re-entry around the right PV drives AF pre-ablation and PVI terminates AF (an example of a responder), with a PV activation flow metric of 22.2%. **(B)** For this simulation, multiple drivers exist in the LA body pre-ablation, and PVI did not terminate AF (an example of a non-responder); the PV activation flow metric is 4.4%. **(C)** Simulated PV activation flow metric is significantly different pre-ablation for PVI responder and non-responder cases (median 21.1 vs. 14.1%, *p* = 0.018, Wilcoxon signed rank).

Preferential pathway analysis was next applied to simulated pre-ablation AF recordings across the cohort of 100 models. AF was initiated in the same way for each model through burst pacing the RSPV, while maintenance mechanisms varied between the models, exhibiting different numbers, stability, and locations of drivers. Figure 5A shows example simulated isopotential maps for AF pre-ablation for a case in which PVI terminated AF, and Figure 5B shows an AF example pre-ablation in which AF continued post-PVI. For the PVI responder case (Figure 5A), re-entry around the right PV drove the AF pre-ablation, with secondary rotational activity and break-up below the left inferior pulmonary vein (LIPV) in an area of fibrotic remodelling. It is challenging to determine the dominant arrhythmia driver from the isopotential maps, but right PV driver dominance was evident on the average optical flow activation map, for which 22% of PV vectors represented activation flow from the PV antra to the LA body. For the PVI non-responder case (Figure 5B), there were multiple drivers in the LA body pre-ablation as well as break-up due to fibrosis, with no clear drivers in the PV regions. The optical flow map is more chaotic, with only 4% of PV vectors representing PV activation flow. Splitting the pre-ablation simulations into PVI responder and non-responder cases results in significantly different PV activation flow metric values, shown in Figure 5C. The results are as follows: median for responder 21.1% vs. non-responder 14.1%; *p* = 0.018 (Wilcoxon signed-rank); sensitivity = 0.79; specificity = 0.58; area under the receiver operating characteristic curve = 0.69.

Fibre field does not have a large impact on simulated acute response or PV activation flow metric in models incorporating fibrotic remodelling (86.7% of model PV activation flow metrics for the two fibre fields were within ±10% of baseline fibre field, see Supplementary Material). These simulations were for the 25 anatomies with patient-specific fibrosis with three different fibre field maps. Overall, this provided a confirmation of the PV activation flow metric for a virtual patient cohort.

### Application to Clinical AF Data: Pathway Analysis and PV Activation Flow Metrics for PVI Responders vs. Non-responders

**Figure 6:**
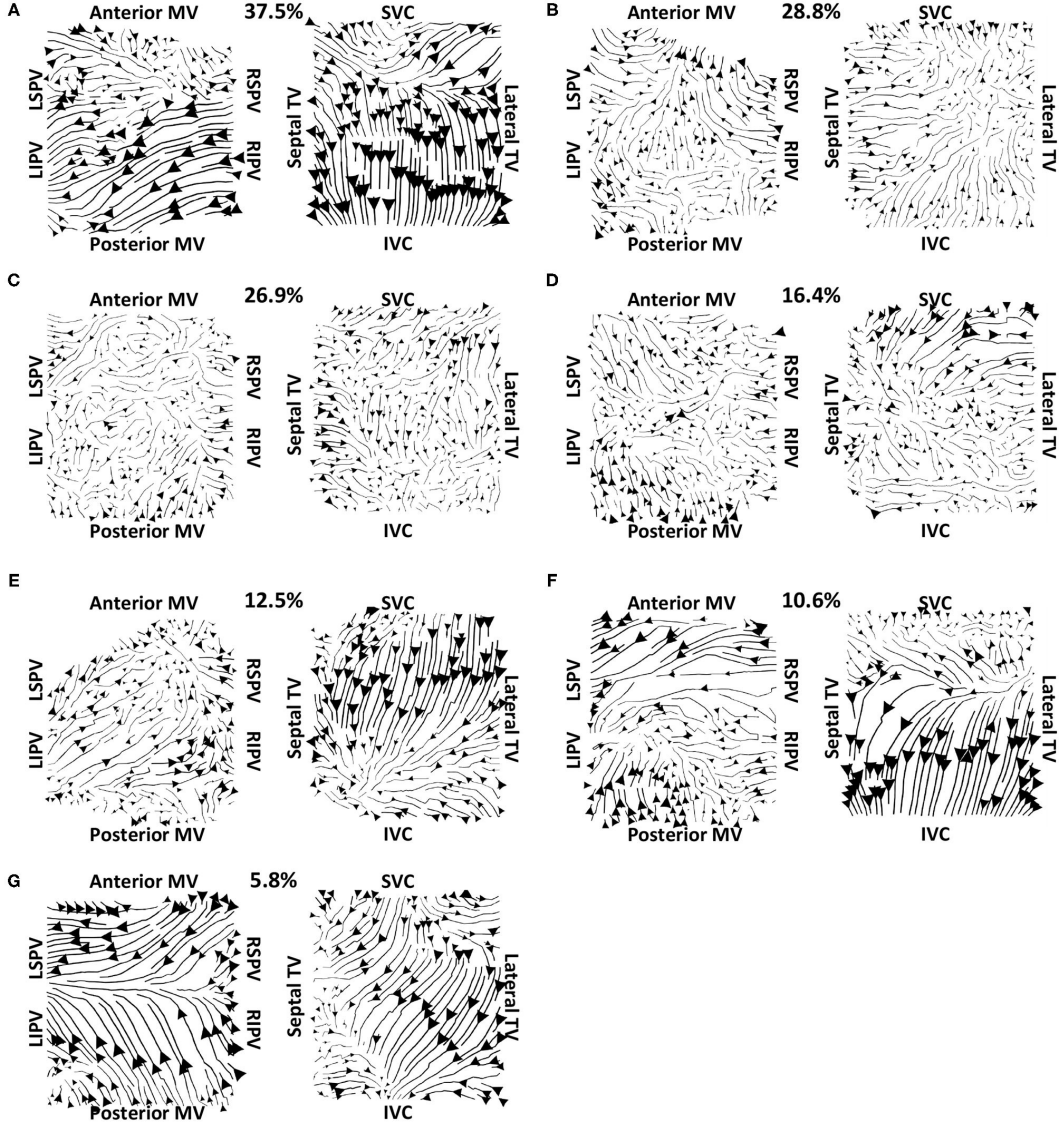
Clinical average vector fields demonstrate activation flow from the PV antra into the atrial body for PVI responder cases pre-ablation. Paths are shown for the left and right atria as streamlines with the magnitude dependent on the magnitude of the underlying average vector field, signifying how often a direction is repeated. The direction of the centre of each streamline is also indicated. Cases are arranged in order of decreasing PV activation flow metric: **(A)** 37.5%, **(B)** 28.8%, **(C)** 26.9%, **(D)** 16.4%, **(E)** 12.5%, **(F)** 10.6%, and **(G)** 5.8%.

**Figure 7:**
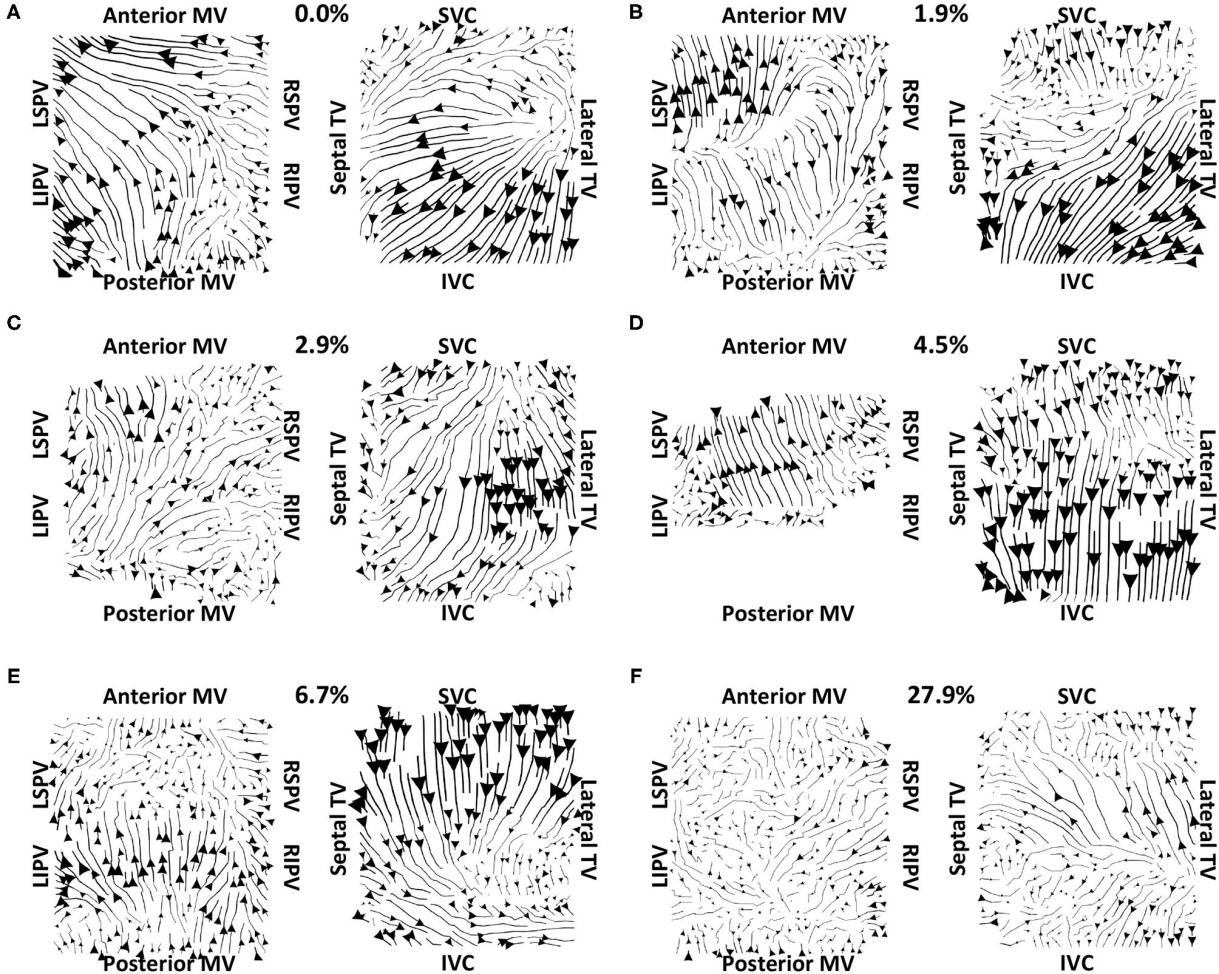
Clinical average vector fields did not demonstrate activation flow from the PV antra into the atrial body for PVI non-responder cases pre-ablation. Visualisation details are as in Figure 6. Cases are arranged in order of increasing PV activation flow metric: **(A)** 0, **(B)** 1.9, **(C)** 2.9, **(D)** 4.5, **(E)** 6.7, and **(F)** 27.9%. For case **(D)**, multiple splines of the LA basket were not in contact and so these were excluded from the analysis.

Vector maps calculated on pre-ablation clinical recordings for PVI responder cases are shown in Figure 6. These maps visually demonstrated preferential flow from the PV antra regions, which were marked as left inferior PV (LIPV), left superior PV (LSPV), right inferior PV (RIPV), and RSPV, into the atrial body. The activation of the atrial body from the PV regions was quantified using the PV activation flow metric. In the pre-ablation cases shown in Figure 6, this was 37.5, 28.8, 26.9, 16.4, 12.5, 10.6, and 5.8%. Conversely, pre-ablation recordings for which PVI ablation did not terminate AF, as shown in Figure 7, did not visually demonstrate a preferential activation flow from the PV antra into the atrial body. Instead, recordings demonstrated a range of repeatability over time: some recordings exhibited the presence of repeated re-entrant activity within the atrial body, while others were more chaotic. The pulmonary vein activation flow metric for these cases pre-ablation are as follows: 0, 1.9, 2.9, 4.5, 6.7, and 27.9%. The average optical flow vector fields with PV activation flow vectors indicated in red are shown for PVI responders in Supplementary Figure 2 and PV non-responders in Supplementary Figure 3.

**Figure 8:**
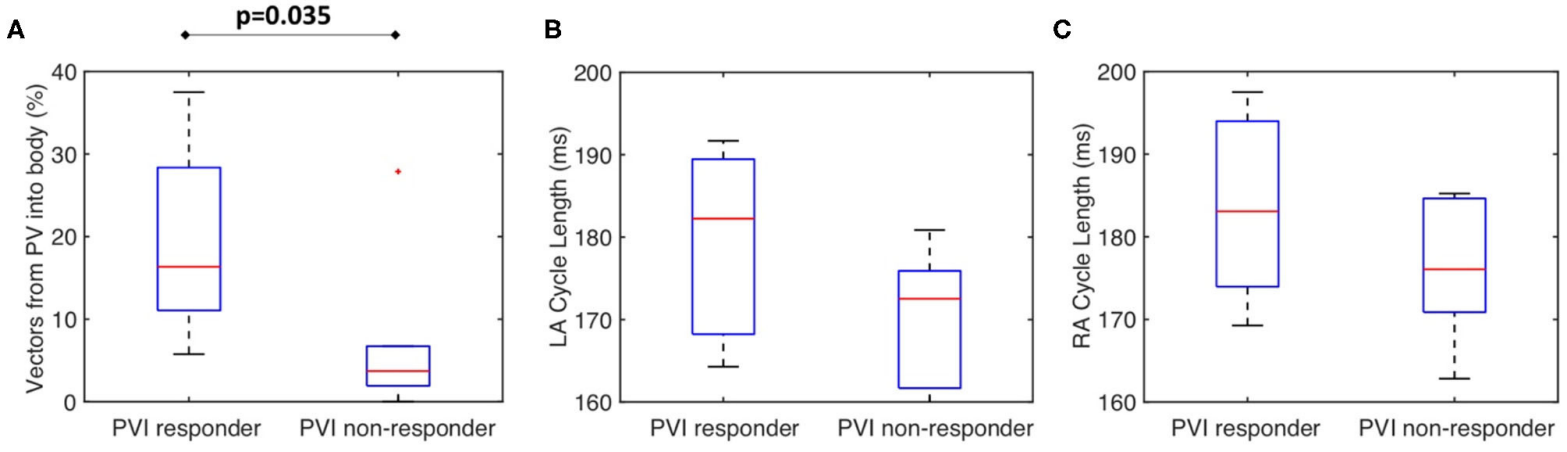
The clinical PV activation flow metric is significantly different for PVI responder and non-responder cases. **(A)** Clinical PV activation flow (%) pre-ablation was significantly higher for PVI responder than PVI non-responder cases (PVI responder: 16.3% vs. non-responder: 3.71%, *p* = 0.035). **(B)** LA cycle length was not significantly different between groups (Wilcoxon signed-rank). **(C)** RA cycle length was not significantly different between groups.

Figure 8A shows that for the clinical dataset, the PV activation flow pre-ablation was significantly higher for PVI responders than PVI non-responder cases. The results are as follows: median 16.3 vs. 3.7%; *p* = 0.035, (Wilcoxon signed-rank); sensitivity = 0.86; specificity = 0.83; area under the receiver operating characteristic curve = 0.86. Other metrics including LA cycle length (Figure 8B: median 182 ms for PVI responder, 173 ms for PVI non-responder) and RA cycle length (Figure 8C: median 183 ms for PVI responder, 176 ms for PVI non-responder) were not significantly different between the PVI responder and non-responder groups.

### Algorithm Sensitivity to Recording Window Choice and Duration Assessed Using Clinical Recordings

**Figure 9:**
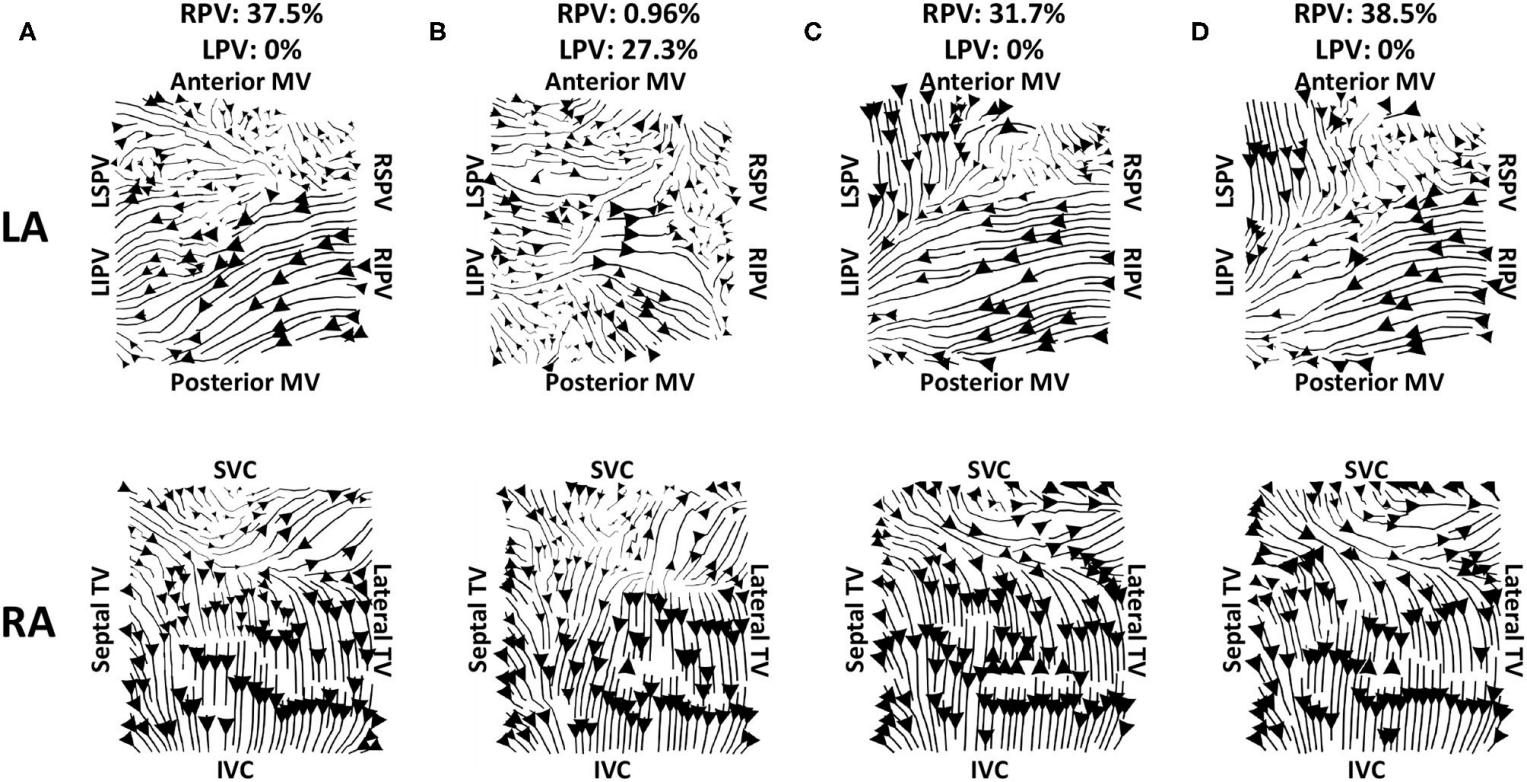
Effects of recording duration on streamlines for example AF episode. Streamlines are shown for 10 s recordings taken from intervals spread through the entire recording. The top row shows the left atrium, and the bottom row shows the right atrium. The streamline plots in **(A)** show the original interval; panels **(C,D)** are visually similar to **(A)**, while the LA for **(B)** demonstrates larger differences. The PV activation flow metrics from the right PV are as follows: **(A)** 37.5%, **(B)** 96%, **(C)** 31.7%, **(D)** 38.5%. There were 9 out of 10 recordings that had no activation flow from the left PV to the LA body, while **(B)** has a PV activation flow metric from the left PV of 27.3%. The start times for these recordings are 0, 6, 125, and 187 s.

To test the effects of recording duration on measured preferential pathways, average optical flow maps were constructed and the PV flow metric was calculated for one clinical case using between 5 and 120 s of data (with 5 s increment). The pulmonary vein flow metric was within a small range of 35–40.4%, and so did not depend on recording duration. We also tested whether the choice of 10 s segment used for analysis from the AF episode affected the PV flow metric by analysing 10 intervals of 10 s spaced at regular intervals through an AF recording. Figure 9 shows example activation streamline maps for different recording segments within a single AF episode. This example AF recording was 217.8 s, and so all intervals represent separate segments with no overlap (approximately 20 s between the start of each interval). For this example, 9 out of the 10 intervals which were assessed had demonstrated visually similar activation, with PV activation flow metric within ±10% of the first interval. Preferential activation pathways were seen from the right PV antra to the LA body for nine of the recordings (for example Figures 9A,C,D), while one interval instead demonstrated flow from the left PV antra to the LA body (Figure 9B).

Comparing the PV activation flow metric for the 10 s segments to the PV activation flow metric for the first window across all clinical cases, showed that 79.2% of PV activation flow metrics for the different windows were within ±10% of the first window (77.1% for PVI responders and 81.7% for PVI non-responders).

### Algorithm Sensitivity to Catheter Size and Contact Tested Through Simulations

**Figure 10:**
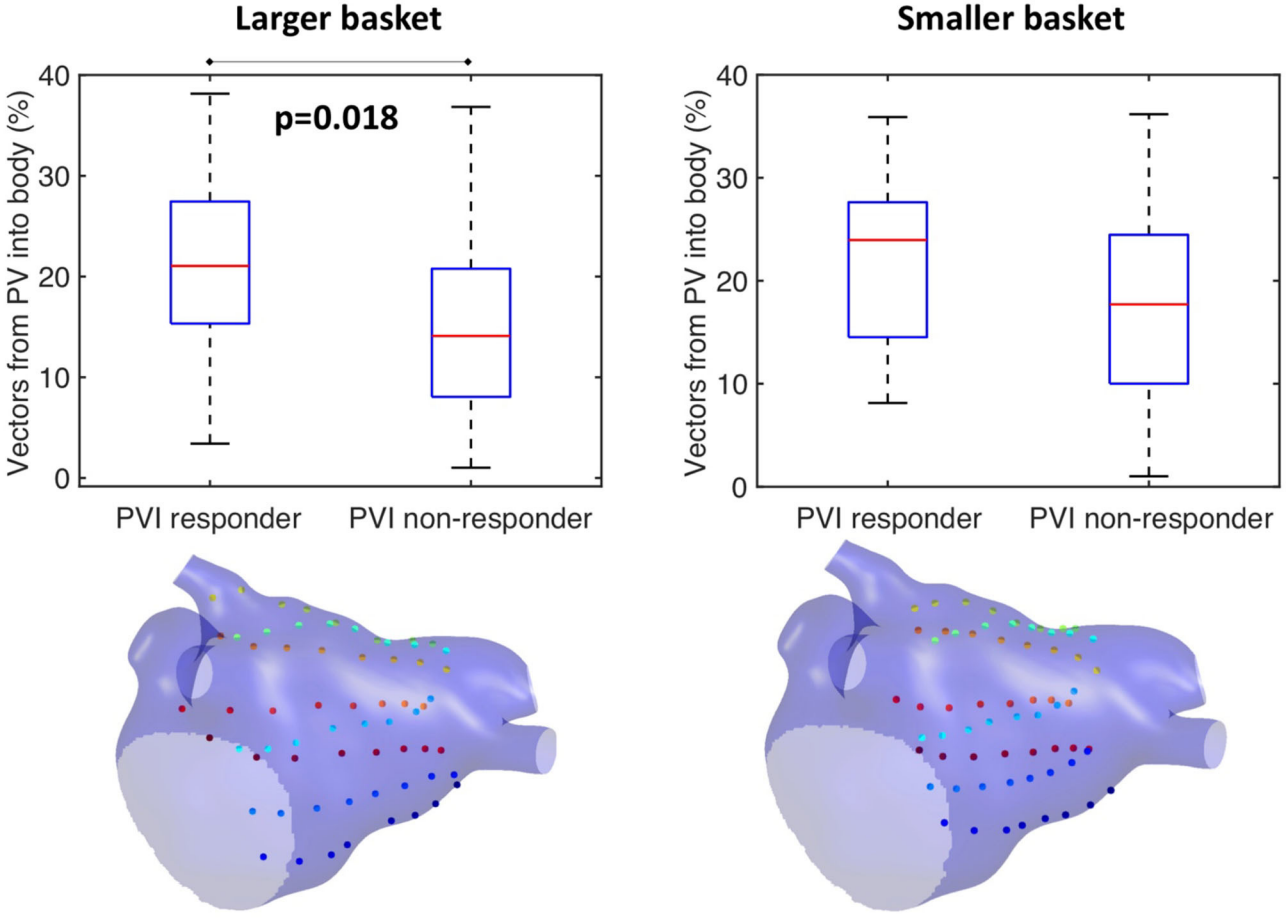
Effects of basket size on preferential pathways. Reducing the basket size changed the significance of the PV flow metric: larger basket: median 21.1 vs. 14.1%, *p* = 0.018, Wilcoxon signed-rank median; smaller basket: 24 vs. 17.7%, not significant.

This study further investigated the effects of basket size and contact on the preferential activation flow calculation and the PV activation flow metric using simulation. Decreasing the basket size from an average spline length of 50 to 35.7 mm for the same simulation set as in section Testing on Simulated AF Data: Pathway Analysis and PV Activation Flow Metrics for PVI Responders vs. Non-responders resulted in a PV activation flow metric difference that is no longer significant (larger basket: median 21.1 vs. 14.1%, *p* = 0.018, Wilcoxon signed-rank median; smaller basket: 24 vs. 17.7%, not significant, see Figure 10). This demonstrated that catheter coverage is important, as well as recording distance from the PVs. The baseline simulated basket size was similar to the smallest of the clinical baskets (simulated: 50 mm; clinically used basket sizes were 48, 60, and 75 mm).

**Figure 11:**
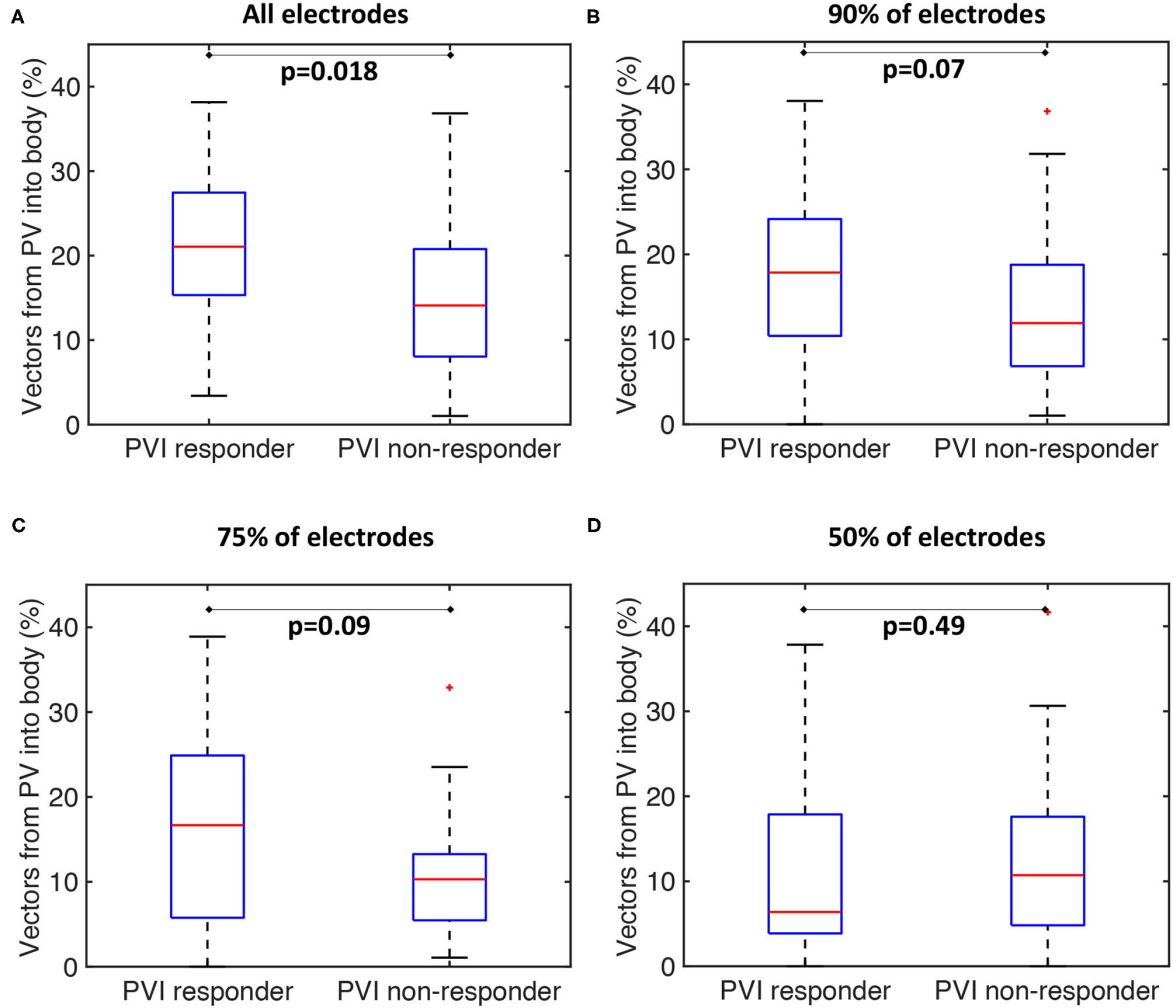
Effects of basket contact on preferential pathways. Effects of poor contact on preferential pathway analysis. PV activation flow metrics are shown for cases with 10–50% of electrodes removed. **(A)** All electrodes: median 21.1 vs. 14.1%, *p* = 0.018, Wilcoxon signed rankmedian. **(B)** 90% of electrodes included: 17.9 vs. 11.9%, *p* = 0.07. **(C)** 75% of electrodes included: 16.7 vs. 10.3%, *p* = 0.09. **(D)** 50% of electrodes included: 6.4 vs. 10.7%, *p* = 0.49.

Our current methodology assumes basket recordings are located on a regular grid. However, for catheter recordings in patients, inter-spline distances vary. The study of Laughner et al. (2016) showed that inter-spline distances exhibit large variations for basket catheters, depending on deployment. In addition, multiple studies have shown that spatial resolution affects the analysis of arrhythmia mechanisms (Roney et al., 2017a). To test these effects on our current analysis, we simulated the effects of removing electrode recordings from the analysis across the 100 atrial models. The effects of poor electrode contact were simulated by randomly removing electrogram recordings from the analysis across all simulations. The percentage of points removed was varied in the range of 10–50%. The pulmonary vein activation flow metric was higher for PVI responder cases than for PVI non-responder cases when the analysis was performed with all electrodes, 90 or 75% of electrodes, although these differences were only significant for the case of all electrodes. It was not possible to differentiate between the PVI responder and PVI non-responder groups when only 50% of electrodes were included in the analysis; shown in Figure 11. We next considered that electrode locations in poor contact are likely to be spatially correlated, and so we considered randomly removing one spline, two splines, or four splines of data from the analysis. These results are shown in Supplementary Figure 4. As for the case of randomly removing electrodes, the PV activation flow metric was higher for PVI responder cases than for PVI non-responder cases when the analysis was performed with all splines, one missing spline or two missing splines. For the case of four missing splines, i.e., only 50% of electrodes included, it was again not possible to differentiate between the two groups.

### Algorithm Sensitivity to Driver Type Tested Through Simulations

**Figure 12:**
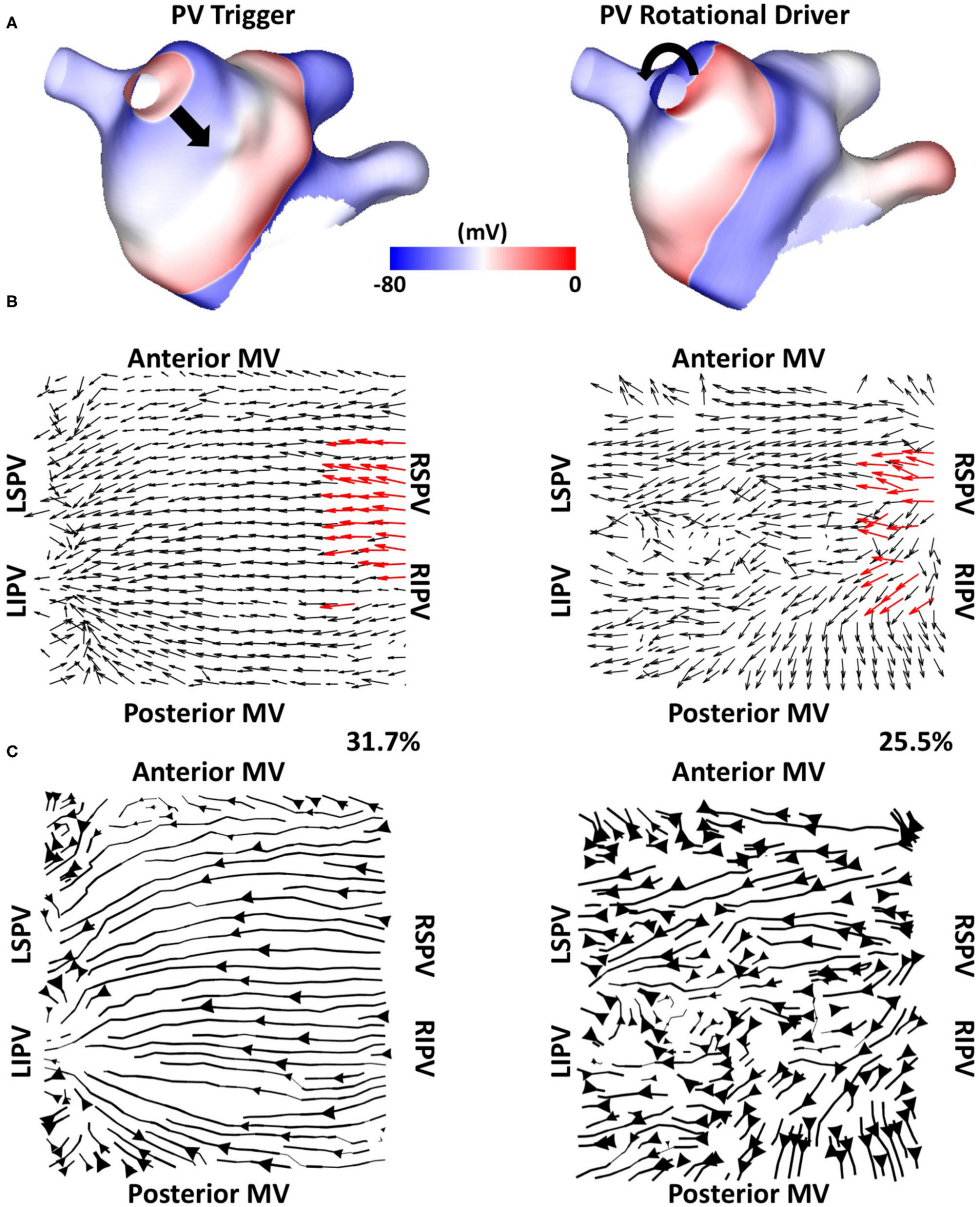
Simulating PV triggers vs. PV rotational drivers. **(A)** Isopotential plots, **(B)** average optical flow activation vector fields and **(C)** activation streamlines are shown for a case sustained by PV triggers (left) and for a PV rotational driver (right).

We tested if the mechanistic source of PV activation flow affected our results. In doing this, simulated cases without fibrosis for which PVI terminated AF (*n* = 6) were selected from the dataset and simulated AF pre-ablation was compared with simulating a repeated PV trigger (through pacing the RSPV at 155 ms for 5 beats). The left side of Figure 12 shows examples (Figure 12A) isopotential maps, (Figure 12B) average optical activation flow vector fields, and (Figure 12C) activation streamlines for a simulation sustained by PV triggers. In this case, preferential activation flow is from the PV antra to the LA body and the PV activation flow metric is 31.7%. The right side of Figure 12 shows the same anatomy but for a simulation where re-entry around the PV antra drives AF, with a PV activation flow metric of 25.5%. The streamline map for the PV trigger case is more organised than the streamline map for the PV rotational driver. Collating the simulations, the PV activation flow metric for PV triggers and PV re-entry cases are not significantly different (mean triggers: 26 ± 8.3 %, mean PV re-entry: 26.5 ± 3.9 %, a paired *t*-test showed not significantly different).

## Discussion

### Main Findings

In this study, we proposed and tested a methodology for detecting preferential activation pathways in patients with persistent AF. We tested the methodology on simulated data and a clinical atrial tachycardia dataset. The technique developed in this study identified patients likely to respond to PVI during the ablation procedure as those with preferential activation flow from the PV antra to the left atrial body. We hypothesised that this activation flow may correspond to the presence of drivers in PV regions. We simulated AF sustained by either PV triggers or by PV rotational drivers to demonstrate that both mechanisms result in a similar PV flow metric. As such, this suggested that isolating the PV antrum is more important in some patients than others when the mechanism for maintaining persistent AF in the atria has preferential pathways using the area around the PVs. Our study could motivate a prospective clinical study to confirm the relationship between preferential flow and long-term PVI outcome.

### Comparison to Other Methodologies

Phase mapping for this dataset did not identify stable rotational activity (Child et al., 2018), suggesting anarchic fibrillation. Instead, analysing these data probabilistically over time identified preferential pathways of activation, indicating a degree of hierarchy.

Our preferential pathway technique has identified repeated structures in the activation patterns. The study of Vandersickel et al. proposed a directed network for identifying repeated activation patterns in the specific case of tachycardia arrhythmias (Vandersickel et al., 2019). Their technique worked on atrial surface meshes rather than projecting to a two-dimensional grid, but this requires a higher resolution data set. Both approaches determine likely circuits of activation. On one hand, the directed network approach in the study of Vandersickel et al. (2019) assumed a stable re-entry circuit, for example, an atrial tachycardia (Van Nieuwenhuyse et al., 2021). On the other hand, our technique also worked for fibrillatory data. As such, we have proposed a novel general tool for identifying critical paths of activation during atrial arrhythmia, which can be applied to atrial macro-reentrant tachycardia (Figure 4) or fibrillation (Figure 6, 7). Similarly, the study of Bellmann et al. (2018) has published an electrographic flow mapping technique to identify electrical sources within the mapping field and classify them as either active or passive. Our technique could be used similarly, but we found no evidence of drivers in the mapping field in this patient cohort. Instead, we utilised the activation flow direction to identify preferential pathways and to assess the arrhythmia, in which we hypothesised that PV triggers or drivers exist for cases in which activation flow occurred from the PV antra to the LA body (Figure 6). Our current methodology assumed global biatrial recordings, and our future work will aim to adapt this technique to work with sequentially acquired data, with a focus on recording at the PV antra.

### Relation to Other AF Mechanisms

Our study demonstrated the importance of activation from the PV in predicting acute PVI response, wherein PVI responder cases had a significantly higher PV activation flow metric pre-ablation than PVI non-responder cases (Figure 8). Similarly, the study of Navara et al. (2018) demonstrated using two mapping methodologies (Rhythmview from Abbott and phase mapping) rotational activity and focal activity in PV antral regions in patients for whom AF terminated during the PVI procedure, which would manifest as preferential activation flow from the PV antra to the LA body. These results agree with our findings. For cases in which activation flow was from the PV antra to the LA body, we found that this was from either the left or right PV, and the utilised PV did not change over time. Previous studies have hypothesised that the smooth PV antra may act as an anchor for rotors (Hocini et al., 2002), which agrees with our simulation studies. Our previous simulation study showed that high PV phase singularity density may indicate the likelihood of a positive PVI response (Roney et al., 2018). Our current study extended our previously proposed metric to one that does not require PV recordings, greatly increasing clinical applicability. Our current simulations suggested that clinical basket sizes are sufficient for PVI ablation outcome prediction using the PV activation flow metric (Figure 10). The simulations also showed that the PV activation flow metric cannot differentiate between PV triggers and PV rotational drivers using LA body recordings alone (Figure 12). Although this distinction is mechanistically important, the PVI outcome is the same in either case.

The examples shown in Figure 6G (PVI responder) and Figure 7F (PVI non-responder) represent outliers. For the PVI responder case (Figure 6G), the optical flow vectors (shown in Supplementary Figure 2G) visually demonstrate flow from the right PV antra to the LA body. However, the magnitudes of these vectors were less than the mean magnitude and so these did not count toward the PV activation flow metric. In this case, the activation flow away from the PV demonstrated a high degree of repeatability. Modifying the threshold magnitude for when to include activation vectors in the PV activation flow metric would increase the value for this outlier. In contrast, the magnitude of repeated directions across the basket device is small for the PVI non-responder case (Figure 7F and Supplementary Figure 3F). For this case, the PV activation flow metric is high even though PV activation flow is not visually evident on the activation streamline map, due to the small vector magnitude in the LA body. It is possible that PV isolation was incomplete for this patient resulting in AF maintenance post-PVI ablation. The other PVI non-responder cases in Figure 7 demonstrated preferential paths in the LA body, distinct from any PV activation flow.

The study of Dharmaprani et al. (2019) characterised AF dynamics through calculating the lifetimes of wavelets and phase singularities demonstrating exponential lifetimes, which was also seen in the study conducted by Child et al. (2018). Our current study demonstrated preferential flow from the PV antra when activation directions were averaged over time. This is compatible with an exponential distribution of phase singularity lifetimes, suggesting that wavefront propagation may demonstrate preferential directions over time.

An alternative AF sustaining mechanism is the presence of re-entry in the RA. For example, the study of Hansen et al. (2015) has demonstrated the presence of intramural re-entry along fibrotic tracks in the human RA. These re-entries were detectable using sub-endocardial mapping for 80% of re-entries, but only for 40% of sub-epicardial re-entries, using FIRM mapping (Zhao et al., 2017). We did not find any evidence of such RA re-entries in this patient cohort using our methodology.

### Simulation Limitations

We used a monolayer model for the simulations in this study. The monolayer is an approximation, like all models, of the atrial activation patterns observed clinically. We chose to use a monolayer model as its complexity reflected the available data. While wall thickness and transmural fibrosis distribution may contribute to atrial arrhythmias and ablation outcomes (Csepe et al., 2017; Roy et al., 2018; Ali et al., 2019), these cannot be reliably measured using standard clinical LGE-MRI. We did not account for these features, and this is an inherent limitation of building models from routine clinical data. The AF simulations in this study were initiated through pacing the RSPV. To investigate the effects of AF initiation protocol on preferential pathways and the PV activation flow metric, we also simulated AF initiation through burst pacing the LSPV or using initial conditions corresponding to four spiral wave re-entries. We found that the AF initiation pacing protocol used affected the preferential pathways and PV activation flow metric, where AF wavefront patterns were generally different for AF initiated using each of the AF initiation protocols for the same model (see Supplementary Figure 5). Despite this, it was still the case that the PV activation flow metric was higher for PVI responders compared with non-responders: for LSPV pacing: 15.6 vs. 5.3% (*p* = 0.06) and for initiation with four spiral wave re-entries: 19.6 vs. 9.6% (*p* = 0.03). These results are presented in the Supplementary Material. Our future work will extend this to systematically investigate the effects of initiation location on preferential pathways and will test this metric for different AF induction protocols (for example, following the studies of Azzolin et al., 2021 and Boyle et al., 2018).

To test whether the PV activation flow metric defined in this study could be used to predict acute PVI response in simulations, we post-processed transmembrane potential signals from AF simulations. Similar to the previous studies (Boyle et al., 2018; Roy et al., 2018; Azzolin et al., 2021), we chose to analyse transmembrane potential signals to eliminate the complexities associated with the effects of wavefront direction on electrogram complexity. However, to demonstrate the full applicability of our pipeline in the clinical environment, it is necessary to also test it on electrogram signals. To test whether the choice of the signal used to calculate preferential pathways affected our simulation results, we compared PV activation flow metrics calculated using transmembrane potential signals to those from unipolar electrogram signals for the LSPV paced dataset. These results are presented in the Supplementary Material where we found that the PV activation flow metric for unipolar signals was similarly higher for PVI responders compared with non-responders, with the same significance value as for the transmembrane potential analysis (*p* = 0.06 for both data types). This suggested the method was not significantly affected by the choice of the input signal, agreeing with our previous study (Roney et al., 2017b). However, these simulations do not include the effects of several clinical complexities, including electrode size, orientation, and noise on the electrogram signals (Potse, 2018).

A further analysis choice or assumption is how to treat data at the mitral and tricuspid valves. We chose to follow the study of Child et al. (2018) and duplicated the anterior MV spline at the posterior side of the grid (using a similar approach for the TV), working on a 9 × 8 grid. This captured that there may be areas where propagation occurred across these splines. An alternative approach was to work on an 8 × 8 grid (Narayan et al., 2012). To test the effects of grid choice, we compared the PV activation flow metric for an 8 × 8 grid to the default 9 × 8 grid analysis. This analysis is presented in the Supplementary Material. Using an 8 × 8 grid, the PV activation flow metric was also significantly higher for the PVI responder cases with a *p*-value of 0.012 (21.4 vs. 14.0%), similar to the results for the 9 × 8 dataset (21.1 vs. 14.1%, *p* = 0.018). The difference between activation flow metrics calculated with or without spline duplication was small, with a mean absolute difference of 1.4%.

### Clinical Limitations

This study has further limitations clinically. We had applied a new method to a limited number of patients in an acute study. The proportion of acute PVI responders is likely to be different in a larger patient population (Verma et al., 2015). A limitation of this study is that patients did not receive the same ablation procedure. All patients had PVI at the start of their ablation, but subsequent ablations were at the discretion of the operator. This was accounted for by assessing acute PVI ablation outcome during the procedure, which while correlated with long-term outcome, is not a surrogate for a long-term outcome (Lim et al., 2015; Kochhäuser et al., 2017; Singh et al., 2017). This study motivated the application of this technique to a larger patient cohort in a prospective trial with standardised ablation procedures and long-term follow-up to determine applicability for general clinical practise. Temporally averaging and spatially coarsening the data to calculate preferential pathways may lose information on individual wavefronts. The technique presented here could be extended to assess the role of the left atrial appendage (Romero et al., 2020), and to identify the intermittent driver or focal regions by analysing shorter recording segments (Gerstenfeld et al., 1992). Future work will compare pathways to atrial fibre structures, including the crista terminalis and septopulmonary bundle (Pashakhanloo et al., 2016; Roney et al., 2020b). Further work should investigate alternative mechanisms for PVI response, for example, by reducing the critical mass of tissue available for fibrillation. The effects of additional ablation lesions on preferential pathways could be investigated in future studies.

## Conclusions

Preferential pathways of activation exist during AF. Our novel technique identified patients that were likely to respond to PVI during an ablation procedure as those with preferential activation flow from the PV antra to the LA body. This flow may correspond to the presence of drivers in the PV regions. We proposed that the metric should be applied in a prospective study, with high-density catheter recordings in the PV at the LA-PV junction, to confirm the relationship between preferential flow, AF mechanisms, and long-term PVI outcome.

## Data Availability Statement

The data analysed in this study is subject to the following licenses/restrictions: The clinical data underlying this article cannot be shared publicly due to privacy reasons. The signal processing codes developed in this article will be shared on github or through openEP (https://openep.io). Requests to access these datasets should be directed to Caroline Roney, caroline.roney@kcl.ac.uk.

## Ethics Statement

The studies involving human participants were reviewed and approved by Regional Ethics Committee (17/LO/0150 and 15/LO/1803). The patients/participants provided their written informed consent to participate in this study.

## Author Contributions

CHR, NC, JG, and SN conceived and designed the study. CHR drafted the manuscript, constructed atrial models, ran the simulations, developed the preferential pathways analysis technique, and analysed the clinical and simulation data. NC, BP, RC, JL, AS, PN, RR, MO'N, CAR, PT, MW, and JG collected, annotated, and analysed the STARLIGHT dataset. IS, JW, and SW collected and segmented atrial imaging data. SN and JG provided the supervision. All authors contributed to the article and approved the submitted version.

## Funding

CHR acknowledges a Medical Research Council Skills Development Fellowship (MR/S015086/1). This study was supported by the UK Engineering and Physical Sciences Research Council (EP/P010741/1, EP/F043929/1, EP/P01268X/1). This study was supported by the Wellcome Trust Center for Medical at King's College London, and the Department of Health via the National Institute for Health Research Biomedical Research Centre award to Guy's & St Thomas' NHS Foundation Trust in partnership with King's College London and King's College Hospital NHS Foundation Trust; the London Medical Imaging and AI Centre for Value-Based Healthcare and the King's College London BHF centre of research excellence. This work was supported by the Wellcome/EPSRC Centre for Medical Engineering [WT 203148/Z/16/Z]. NC was funded by an education grant from St Jude Medical (Abbott).

## Conflict of Interest

JL and AS were employed by Boston Scientific Corp. The remaining authors declare that the research was conducted in the absence of any commercial or financial relationships that could be construed as a potential conflict of interest.

## Publisher's Note

All claims expressed in this article are solely those of the authors and do not necessarily represent those of their affiliated organizations, or those of the publisher, the editors and the reviewers. Any product that may be evaluated in this article, or claim that may be made by its manufacturer, is not guaranteed or endorsed by the publisher.
