# Supplementary material for: Time-Averaged Wavefront Analysis Demonstrates Preferential Pathways of Atrial Fibrillation, Predicting Pulmonary Vein Isolation Acute Response
Source: Front Physiol. 2021 Sep 27;12:707189. doi: 10.3389/fphys.2021.707189 (PMC8503618; doi:10.3389/fphys.2021.707189)
Supplement: Supplementary file 1 [file Data_Sheet_1.pdf]

# *Supplementary Material*

## 1 Methodology: Electrogram Processing to calculate the normalized filtered derivative and phase

Unipolar electrogram signals were processed to calculate a normalised derivative signal and a phase signal using a sequence of steps, shown in **Supplementary Figure 1**. First, QRS subtraction was applied to unipolar electrograms to remove any ventricular artefacts from the signals following the median template technique of (Shkurovich et al., 1998). Following QRS subtraction, unipolar electrograms were differentiated, and this derivative signal was capped at zero to prevent the algorithm from assigning activation to points of positive (rather than steepest negative) slope. The capped derivative signal was filtered using a sequence of filters typically used prior to dominant frequency analysis in order to make the signal more sinusoidal (Ng et al., 2007). Specifically, the signal was bandpass filtered from 40-250 Hz (butterworth, order 3), full-wave rectified, and low pass filtered at 10 Hz (butterworth, order 8). This sequence of filters was originally presented and tested by Botteron et al. (Botteron and Smith, 1995)(Castells et al., 2014). This filtering used the *butter* and *filtfilt* functions in Matlab to perform bidirectional processing and cancel phase shifts.

Calculation of the normalised derivative signal and unipolar phase followed our previously published methodology (Roney et al., 2017b). For simulated transmembrane potential signals, we did not filter the signals, and started the analysis at this point. In brief, an adaptation of the pseudo empirical mode decomposition technique of Bray and Wikswo was used to create a zero-mean sinusoidal signal with activation times at constant normalised derivative value (Bray and Wikswo, 2002a). Maxima in the filtered derivative signal were assigned using a moving window of length equal to 90% of the median cycle length (estimated using the dominant frequency), and minima tagged between each pair of maxima. Maxima and minima cubic splines were calculated and used to normalise the signal. This normalized signal was raised to the power of six to dampen low-amplitude untagged deflections, and capped at one to correct any segments of the signal that were above the maxima line. This signal was used as the normalized filtered derivative signal throughout our analysis. We tested the effects of parameter choices, including the window length for tagging maxima, in our previous study (Roney et al., 2017b).

To calculate phase, a straight mean was removed from the normalised derivative signal, and the resulting signal was plotted against its Hilbert transform, as a phase-space plot. The angle around this trajectory gave the phase angle.

These steps are shown in **Supplementary Figure 1**.

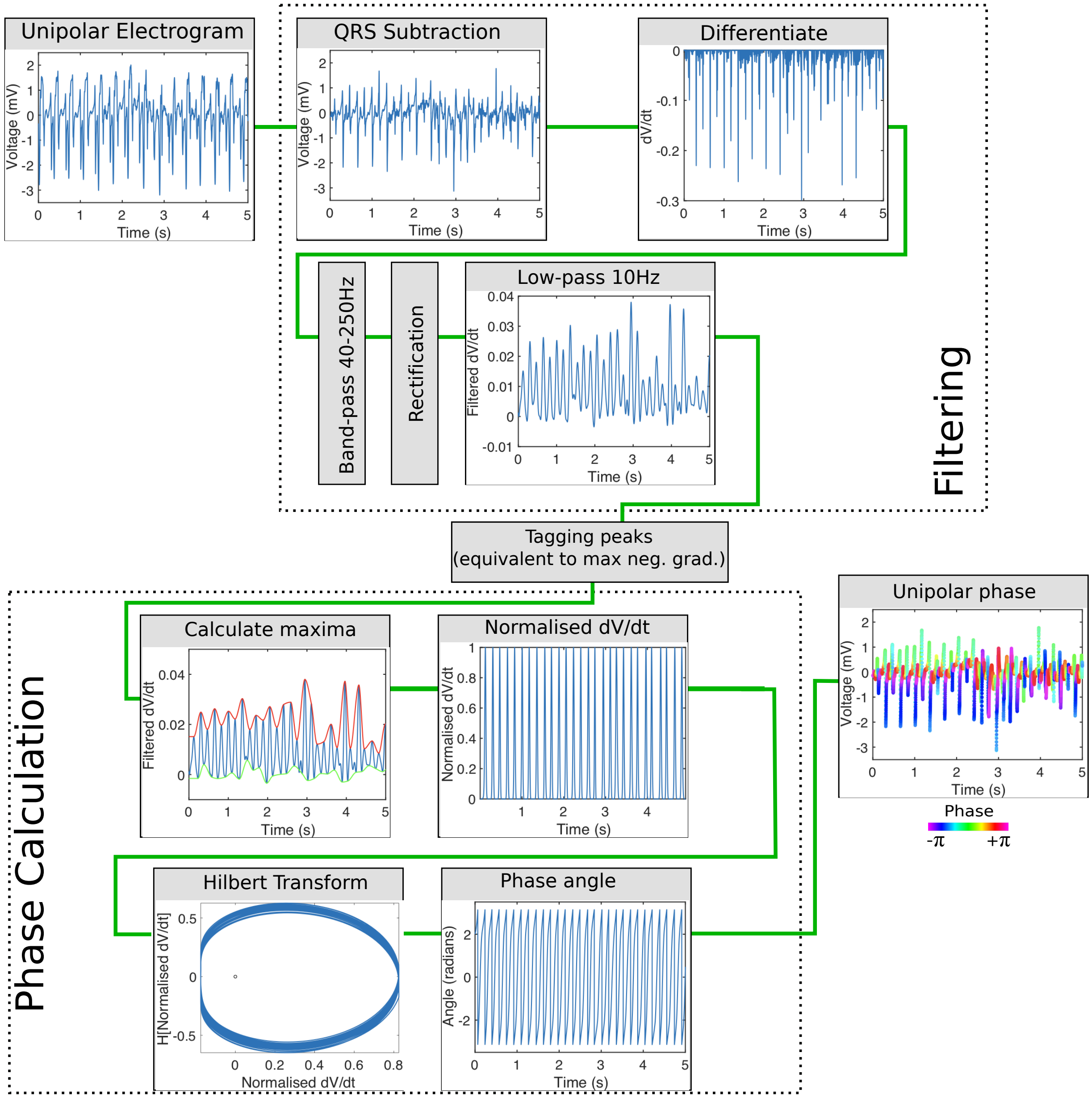


**Figure 1: Methodology used for calculating the normalised derivative signal and phase from unipolar electrograms.**
*Unipolar electrograms were processed using a QRS subtraction technique followed by differentiation and a sequence of filters, as described in the text. Moving maxima and minima splines were calculated for the filtered derivative signal and used to create a normalised signal. This normalised filtered derivative signal was calculated for each unipolar electrogram signal.*

## 2 Methodology: Normalised derivative mapping

Both unipolar electrogram phase and the normalised derivative signal were calculated for each of the 64 electrodes on each basket catheter. These data were then displayed in a 9-by-8 arrangement, with the posterior MV at the bottom of the figure, the anterior MV at the top, and the left PV (lateral wall) and right PV (septal wall) on the left and right of the figure respectively. Correspondingly for the

RA, the IVC was displayed at the bottom of the figure, SVC at the top, septal TV on the left, and lateral TV on the right. These data were linearly interpolated to a higher resolution grid with an additional two points between each two points on the original grid. Excluded recordings were replaced by interpolated values. We used an exponential mapping technique for phase interpolation to prevent discontinuities across the phase angle branch cut (Roney et al., 2017a).
Phase singularities were identified on this regular grid using the topological charge technique of Bray and Wikswo (Bray and Wikswo, 2002b), and tracked over time; subject to distance and temporal thresholds, following our previous methodology (Roney et al., 2018).

## 3 Results: Optical flow activation pathway maps

**Supplementary Figure 2** shows average optical flow vector fields and streamlines for PVI responder cases, corresponding to **Figure 6** of the main manuscript. **Supplementary Figure 3** shows the equivalent plot for PVI non-responders, corresponding to **Figure 7** of the main manuscript.

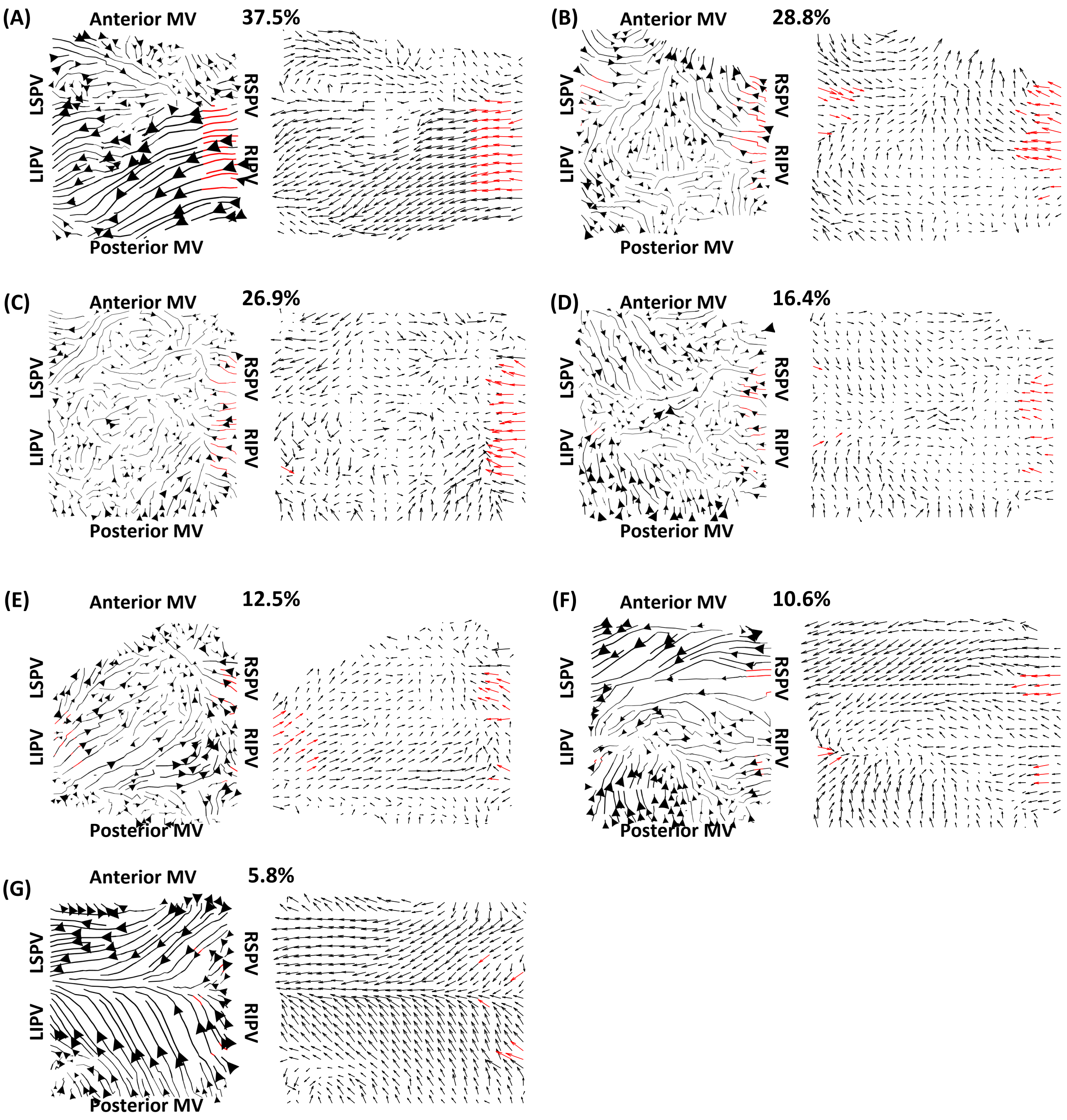


***Figure 2: Clinical average optical flow vector fields and streamlines for PVI responder cases pre-ablation.***

*PV activation flow vectors are shown in red. PV activation flow percentages are given as the maximum of the left PV activation flow and right PV activation flow.*

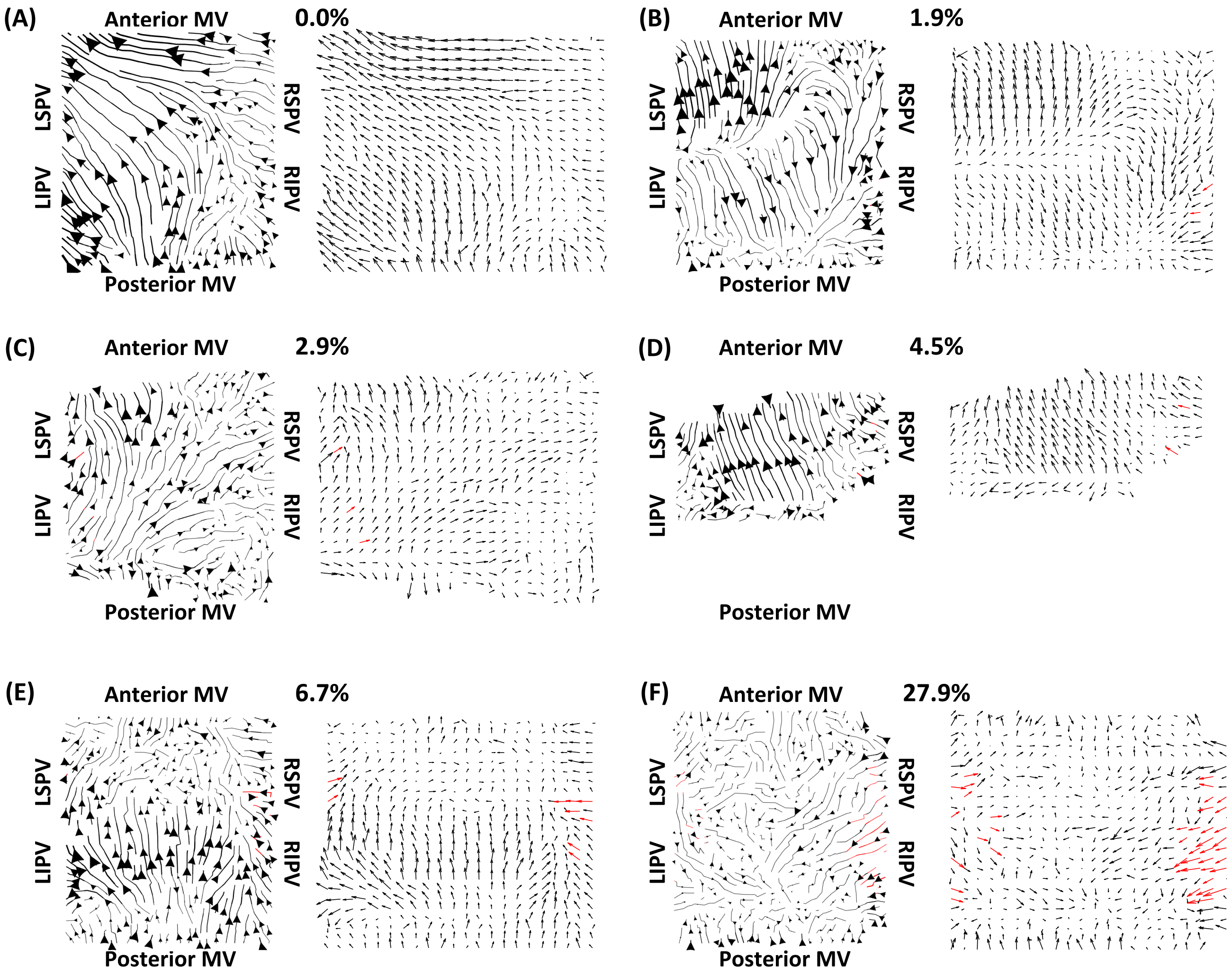


***Figure 3: Clinical average optical flow vector fields and streamlines for PVI non-responder cases pre-ablation.***

*PV activation flow vectors are shown in red.*

## 4 Results: Effects of anisotropy fibre field on pathways

Comparing the acute response of the two different fibre fields to the baseline fibre field, sensitivity was 0.80 for each field, and accuracy was 0.71 for the DTMRI fibre field and 0.76 for the average fibre field. The average absolute difference in PV activation flow metric compared to the baseline model was 5.18 ± 3.74% for the DTMRI fibre field and 5.84 ± 4.18% for the average fibre field. The PV activation flow for the two fibre fields was within ±10% of that of the baseline fibre field for 86.7% of the models. This demonstrates that fibre field does not have a large impact on simulated acute response or PV activation flow metric for models incorporating fibrotic remodelling.

## 5 Results: Effects of basket contact on preferential pathways

We simulated the effects of randomly removing one spline, two splines or four splines of data from the analysis. These results are shown in **Supplementary Figure 4**. Similar to the case of randomly removing electrodes (main manuscript **Figure 11**), the PV activation flow metric was higher for PVI responder cases than for PVI non-responder cases when the analysis was performed with all electrodes, one missing spline or two missing splines. For the case of 4 missing splines (i.e. only 50% of electrodes included), it was again not possible to differentiate between the two groups.

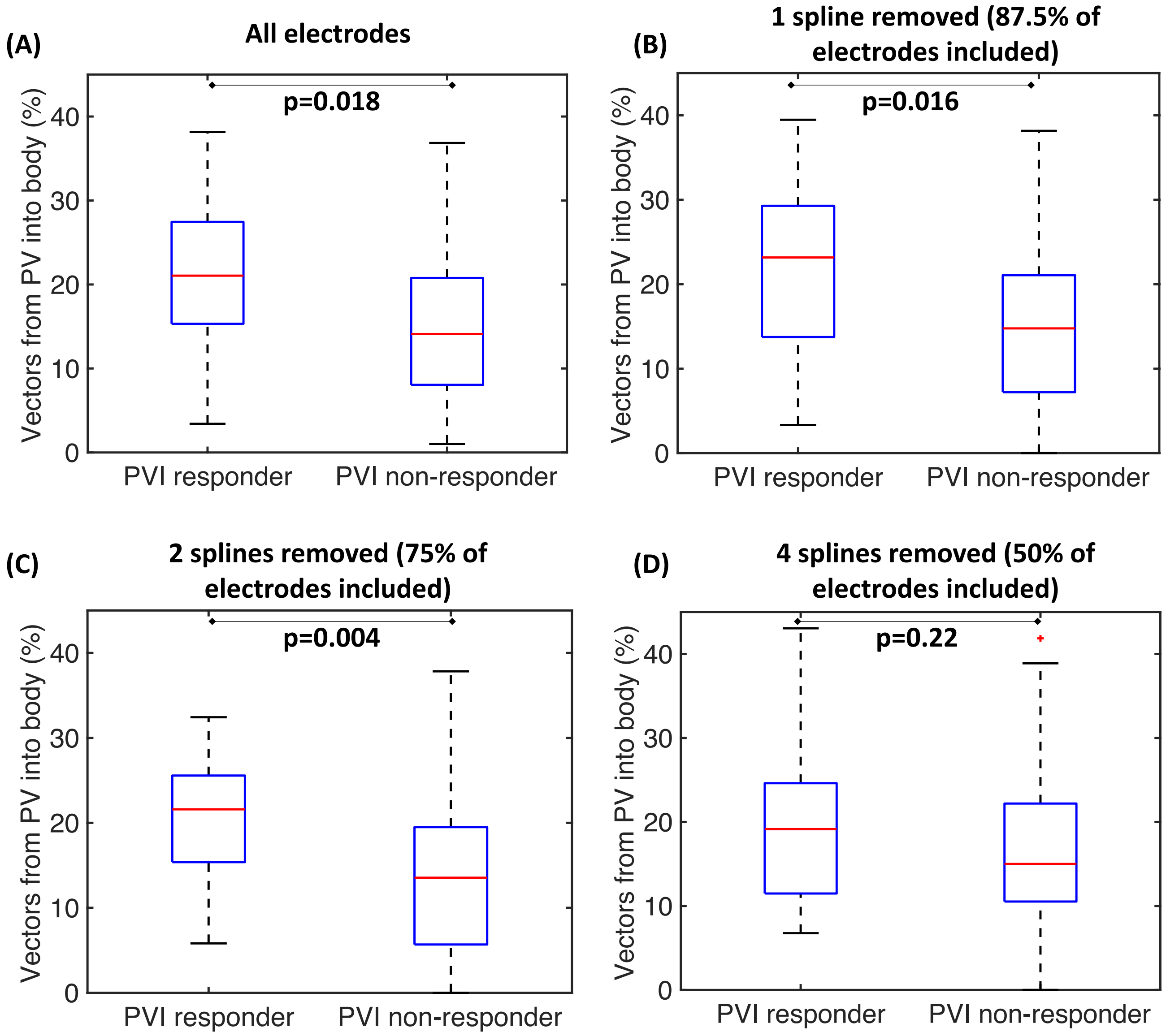


***Figure 4: Effects of basket contact on preferential pathways.***

*Effects of poor contact on preferential pathway analysis. PV activation flow metrics are shown for cases with 1 spline, 2 splines or 4 splines removed. (A) All electrodes: median 21.1% vs 14.1%, p=0.018, Wilcoxon signed rankmedian. (B) One spline removed: 23.2% vs 14.8%, p=0.016. (C) Two splines removed: 21.6% vs 13.5%, p=0.004. (D) Four splines removed: 19.1% vs 15.0%, p=0.22.*

## 6 Results: Effects of AF initiation pacing protocol on preferential pathways

To test the effects of AF induction pacing protocol on our findings, we simulated two additional AF induction pacing protocols – one with pacing from the LSPV and one with four spiral wave re-entries – and applied these to the 25 anatomies with patient-specific fibrosis. For one simulation set, AF was induced through burst pacing the left superior pulmonary vein (LSPV), again at a cycle length of 155ms for ten beats following sinus rhythm. For the second simulation set, AF was initiated by setting initial conditions that corresponded to four spiral wave re-entries, following our previous study (Roney et al., 2020a). These initial conditions were an activation time field with two Archimedean spirals on each of the posterior and anterior walls, which were assigned to each anatomy using the universal atrial coordinate system (Roney et al., 2019).

The AF initiation pacing protocol used affected the preferential pathways and PV activation flow metric, where AF wavefront patterns were generally different for AF initiated using each of the AF initiation protocols for the same model; an example is shown in **Supplementary Figure 5**. Despite this, it was still the case that the PV activation flow metric was higher for PVI responders compared to non-responders: for LSPV pacing: 15.6% vs 5.3% (p=0.06) and for initiation with four spiral wave re-entries: 19.6% vs 9.6% (p=0.03).

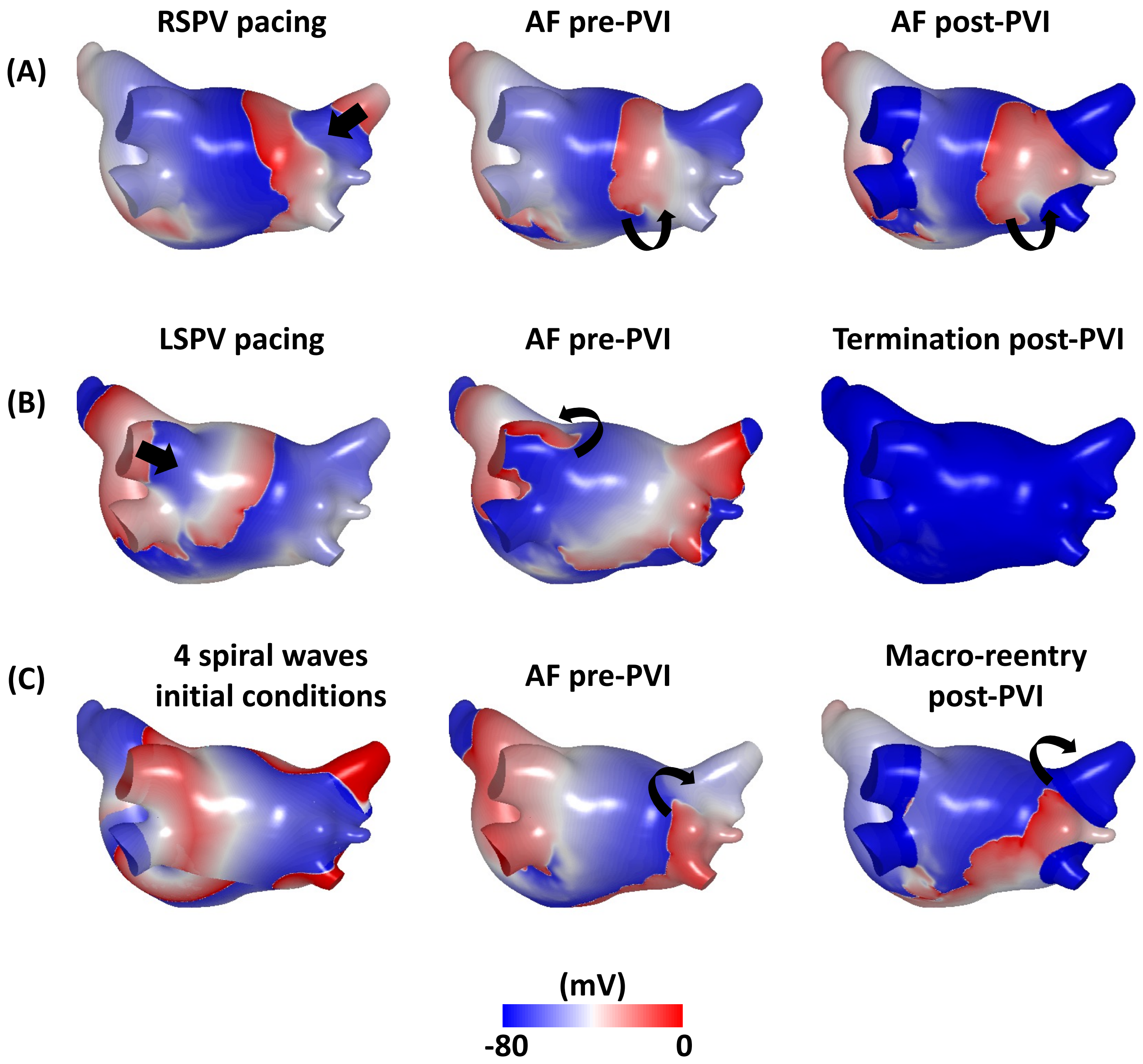


***Figure 5: Effects of AF initiation pacing protocol on preferential pathways.***
*Transmembrane potential maps for arrhythmias initiated by: (A) pacing the RSPV, (B) pacing the LSPV, (C) setting initial conditions corresponding to four spiral wave re-entries. The black arrows indicate wavefront propagation direction. The left column shows the pacing protocol used for AF initiation. The middle column is a snap shot typical of the AF wavefront propagation patterns for this model. The right column shows the outcome after simulated PVI ablation. In the case of RSPV pacing in (A), there are two re-entries in the body of the left atrium, and this case has a PV activation flow metric of 10.0%. For LSPV pacing in (B), there is a re-entry around the LSPV throughout the simulation, which drives AF, and the PV activation flow metric is 33.3%. For (C), there is a re-entry around the RSPV throughout the simulation, which drives the AF, and the PV activation flow metric is 22.5%. For RSPV pacing, AF is sustained post-PVI ablation; for LSPV pacing, AF terminates*

*post-PVI ablation; for the four spiral wave re-entry initiation, AF converts to macro-reentry post-PVI ablation.*

## 7 Results: Effects of signal type (transmembrane potential or electrogram) on PV activation flow metric

We used the LSPV simulation dataset to test whether the choice of the signal used to calculate preferential pathways affected our simulation results by comparing PV activation flow metrics calculated using transmembrane potential signals to those from unipolar electrogram signals. For the unipolar electrogram signals, the PV activation flow metric was again higher for PVI responders compared to non-responders, with the same significance value as for the transmembrane potential analysis (p=0.06 for both data types).

## 8 Results: Effects of grid choice on PV activation flow metric

To test the effects of duplicating the anterior MV spline at the posterior side of the grid following (Child et al., 2018), we calculated the PV activation flow metric with or without this duplication, by comparing a 9-by-8 to an 8-by-8 grid. This analysis was performed across the baseline simulation set (100 atrial models). Using an 8-by-8 grid the PV activation flow metric was significantly higher for the PVI responder cases with a p value of 0.012 (21.4% vs 14.0%), compared to p=0.018 for the 9-by-8 dataset (21.1% vs 14.1%). The difference between activation flow metrics calculated with or without spline duplication was small, with a mean absolute difference 1.4%.

## 9 References


Botteron, G. W., and Smith, J. M. (1995). A technique for measurement of the extent of spatial organization of atrial activation during atrial fibrillation in the intact human heart. *IEEE Trans. Biomed. Eng.* 42, 579–586. doi:10.1109/10.387197.

Bray, M.-A., and Wikswo, J. (2002a). Considerations in phase plane analysis for nonstationary reentrant cardiac behavior. *Phys. Rev. E* 65, 51902. doi:10.1103/PhysRevE.65.051902.

Bray, M.-A., and Wikswo, J. P. (2002b). Use of topological charge to determine filament location and dynamics in a numerical model of scroll wave activity. *IEEE Trans. Biomed. Eng.* 49, 1086–93. doi:10.1109/TBME.2002.803516.

Castells, F., Cervigón, R., and Millet, J. (2014). On the preprocessing of atrial electrograms in atrial fibrillation: understanding Botteron's approach. *Pacing Clin. Electrophysiol.* 37, 133–43. doi:10.1111/pace.12288.

Ng, J., Kadish, A. H., and Goldberger, J. J. (2007). Technical considerations for dominant frequency analysis. *J. Cardiovasc. Electrophysiol.* 18, 757–64. doi:10.1111/j.1540-8167.2007.00810.x.

Roney, C. H., Cantwell, C. D., Bayer, J. D., Qureshi, N. A., Lim, P. B., Tweedy, J. H., et al. (2017a).

Spatial Resolution Requirements for Accurate Identification of Drivers of Atrial Fibrillation. *Circ. Arrhythmia Electrophysiol.* 10. doi:10.1161/CIRCEP.116.004899.

Roney, C. H., Cantwell, C. D., Qureshi, N. A., Chowdhury, R. A., Dupont, E., Lim, P. B., et al. (2017b). Rotor Tracking Using Phase of Electrograms Recorded During Atrial Fibrillation. *Ann. Biomed. Eng.* 45, 910–923. doi:10.1007/s10439-016-1766-4.

Roney, C. H., Ng, F. S., Debney, M. T., Eichhorn, C., Nachiappan, A., Chowdhury, R. A., et al. (2018). Determinants of new wavefront locations in cholinergic atrial fibrillation. *Europace* 20, III3-III15. doi:10.1093/europace/euy235.

Shkurovich, S., Sahakian,  a V, and Swiryn, S. (1998). Detection of atrial activity from high-voltage leads of implantable ventricular defibrillators using a cancellation technique. *IEEE Trans. Biomed. Eng.* 45, 229–34. doi:10.1109/10.661270.
